# Study of sex-biased differences in genomic profiles in East Asian hepatocellular carcinoma

**DOI:** 10.1007/s12672-024-01131-9

**Published:** 2024-07-09

**Authors:** Chung-Yu Huang, Kien-Thiam Tan, Shiu-Feng Huang, Yen-Jung Lu, Yeh-Han Wang, Shu-Jen Chen, Ka-Po Tse

**Affiliations:** 1 ACT Genomics Co., Ltd., Taipei, Taiwan; 2 Anbogen Therapeutics, Inc., Taipei, Taiwan; 3 Core Pathology Lab, Institute of Molecular and Genomic Medicine, National Health Research Institutes, Miaoli, Taiwan; 4 Department of Pathology, Fu Jen Catholic University Hospital, Fu Jen Catholic University, New Taipei City, Taiwan; 5 School of Medicine, College of Medicine, Fu Jen Catholic University, New Taipei City, Taiwan

**Keywords:** Hepatocellular carcinoma (HCC), Sex disparity, East Asian, Prognosis, Precision medicine

## Abstract

Hepatocellular carcinoma (HCC) is characterized by a notable sex disparity in incidence and tumor aggressiveness. Revealing differences in genetic landscapes between male and female HCCs may expand the understanding of sexual disparities mechanisms and assist the development of precision medicine. Although reports on the sex disparity of HCC are accumulated, studies focusing on sex-related biomarkers among Asian populations remain limited. Here, we conducted a comprehensive genomic profiling analysis to explore differences between male and female patients within a cohort of 195 Taiwanese HCC patients. We did not detect any sex-biased genomic alterations. However, when our investigation extended to the TCGA dataset, we found higher frequencies of gene copy gains in *CCNE2* and mutations in *CTNNB1* and *TP53* among male patients. Besides, we further evaluated the associations between genomic alterations and patients’ prognosis by sex. The results showed that female patients harboring tumors with *STAT3* gain and alterations in the JAK–STAT pathway displayed a poor prognosis. These two factors remained independently associated with unfavorable prognosis even after adjusting for the patient’s age and stage characteristics (Hazard ratio = 10.434, 95% CI 3.331–32.677, P < 0.001; Hazard ratio = 2.547, 95% CI 1.195–5.432, *P* = 0.016, respectively). In summary, this study provides valuable insights into understanding sex disparity in HCC in the East Asian population. Validation through larger cohorts and extensive sequencing efforts is warranted.

**Supplementary Information:**

The online version contains supplementary material available at 10.1007/s12672-024-01131-9.

## Introduction

For decades, sex disparity in incidence and prognosis has been evident in various types of human diseases, including non-reproductive cancers. A higher incidence of several types of cancer was reported in males, such as liver, esophagus, and bladder cancers [1, 2]. In contrast, subtypes of lung cancer and thyroid cancer are more prevalent in females [3]. In most cancers, men experience poorer prognoses than women and female patients show more sensitivity than males to the toxicity of different chemotherapeutic drugs [4–6]. However, despite sex being known as one of the most critical factors affecting incidence, aggressiveness, responses to treatment, and adverse drug reactions in cancer patients, the sex of the patients is less considered during drug development or clinical decision-making [2, 7].

Hepatocellular carcinoma (HCC), which accounts for 75–85% of all primary liver cancer [8], is one of the malignant solid tumors known for noticeable sex disparity [9]. The incidence ratios of males to females vary between 2:1 to 4:1 across different populations, depending on the geographic region [10, 11]. In Taiwan, the age-adjusted incidence rate of HCC is approximately three times higher in males (male: female ratio, 40.95: 15.44 per 100,000 persons = 2.65:1) [12]. Moreover, studies from some populations indicated that women were older at HCC diagnosis and had a better prognosis than men [13–15]. However, reports from other areas showed no difference in survival by sex [16, 17].

Efforts have been spent to unravel the mechanisms behind the observed differences in HCC between sexes. A comprehensive literature analysis reveals a multifaceted interplay of factors that contributes to this sex disparity [18–22]. These factors include the expression levels of genes encoded on the sex chromosome, the influence of sexual hormones, variation in lifestyles, immune responses, and sex-specific dysregulation of gene expression and mutational profiles. For instance, the X chromosome stands out for its enrichment of sex-biased genes and housing many immune-related genes encoding proteins that regulate both innate and adaptive immunity [23, 24]. Estrogen, which plays a protective role in women, has been associated with lower infection rates of hepatitis B virus (HBV) [25]. This correlation is supported by the observation that HBV-related HCC occurs more frequently in men during the reproduction period [26], while a significant increase in HCC incidence has been noted in postmenopausal women [19].

On the other hand, studies also found that male with HBV infection has a high incidence of HCC due to the male hormone effect. The androgen receptor promotes hepatitis B virus (HBV)-induced hepatocarcinogenesis through modulation of HBV RNA transcription [20], and male HBV carriers with higher testosterone levels were also reported to have an increased risk of HCC [27, 28]. Manieri et al. proposed that reduced production of adiponectin after puberty or in an obese state may increase the risk of HCC in males [29].

With the advent of next-generation sequencing (NGS), several genomic projects presented by The Cancer Genome Atlas (TCGA) and other groups have tried to elaborate on the differences in molecular signatures between male and female HCC [18, 24]. Results demonstrated that male HCC patients harbored higher levels of somatic mutation, copy number variation (CNV), DNA methylation, mRNA, and miRNA expression [24]. The *CTNNB1* gene showed significantly higher mutation frequency in male HCC [24] as more BRCA1-associated protein 1 (*BAP1*) gene mutations were reported in female HCC [30]. Male patients displayed enriched activation of PI3K, PI3K/AKT, FGFR, EGFR, NGF, GF1R, Rap1, DAP12, and IL-2 signaling pathways, while female patients showed PPAR pathway enrichment [18]. Besides genomic variants, some protein expressions also showed bias between sexes. For example, one study demonstrated that *CYP39A1*, a gene specifically expressed in the liver and has higher expression in females, was significantly reduced in 90% of HCC patients. Further investigation revealed that *CYP39A1* exerts a tumor-suppressive effect by inhibiting the transcriptional activity of c-Myc [31].

Considering that most studies are predominantly based on populations of European ancestry [8, 11, 32], the Asian population was under-represented. Here, we aimed to explore the genomic landscapes between male and female HCC patients in the Taiwanese population. Also, we wanted to investigate the sex-specific biomarkers for HCC prognosis prediction.

## Materials and methods

### Study population and design

Purified DNA of archived HCC specimens was collected from the Taiwan Liver Cancer Network (TLCN), a representative nationwide biobank established in 2005 [33]. This biobank contains samples and demographics of liver cancer patients from Taiwan’s five major medical centers. The National Health Research Institutes Research Ethics Committee approved this study IRB (EC1080601-F-E). It waived the requirement of informed consent because of the retrospective nature of our study using de-identified data. The study was conducted following the Declaration of Helsinki.

One hundred and ninety-five specimens we used in this study were collected between Sep 2005 and May 2011. The latest follow-up date was 1 July 2019, with a median overall survival (OS) of 80 months (ranging from 0.7 to 165 months). All tissue was collected by surgery without pretreatment. The demographic characteristics include age, sex, stage of disease, history of alcohol/smoking, cirrhosis, HBV/HCV infection history, date of diagnosis, date of sample collection, relapse, and latest follow-up were retrieved from the biobank database.

### The cancer genome atlas database

The Liver Hepatocellular Carcinoma dataset, TCGA Firehose Legacy, was obtained from the cBioPortal platform (https://www.cbioportal.org/). Out of the initial pool of 379 liver cancer cases, a total of 366 cases of HCC were selected for analysis. Thirteen samples, which included cases of hepatocellular carcinoma plus intrahepatic cholangiocarcinoma, fibrolamellar carcinoma, and liver cancer without specified subtypes, were excluded from the study. Consequently, our analysis focused on 366 samples, with 362 having single nucleotide variation (SNV) profiles and 359 featuring copy number variation (CNV) profiles for data analysis.

### Targeted next-generation sequencing (NGS)

One hundred and ninety-five samples DNA were sequenced using ACTOnco^®^(ACT Genomics Co., Ltd., Taipei, Taiwan), a comprehensive panel targeting 440 cancer-related genes with whole exon coverage. The experimental procedures were as previously described [34]. In brief, genomic DNA was amplified using four pools of primer pairs targeting the coding exon regions of targeted genes. Amplicons were ligated with barcoded adaptors. Samples passed the quality control were subsequently conjugated with sequencing beads by emulsion PCR and enriched using the Ion Chef system (Thermo Fisher Scientific, Massachusetts, US) according to the Ion PI Hi-Q-Chef Kit protocol (Thermo Fisher Scientific, Massachusetts, US). The amplified product was sequenced on the Ion Proton or Ion S5 sequencer (Thermo Fisher Scientific, Massachusetts, US). The test provided a mean coverage of ≥ 500× and target base coverage at 100× ≥ 85%.

### Variant calling and annotation

The raw reads generated were mapped to the hg19 reference genome using the Ion Torrent Suite (version 4.2). Coverage depth was calculated using the Torrent Coverage Analysis plug-in. Variants reported in the Genome Aggregation database (version 2.0.2) with ≥ 1% minor allele frequency (MAF) were considered as polymorphisms and excluded from the following analyses.

Single nucleotide variants (SNVs), multi-nucleotide variants (MNVs), and small insertion/deletion (INDELs) were identified using the Torrent Variant Caller plug-in (version 4.2). Using ClinVar, COSMIC v.70, and the Genome Aggregation database (version 2.0.2) as references, VEP (Variant Effect Predictor; version 78) was used to annotate every variant. Variants with coverage ≥ 25, allele frequency ≥ 5%, and actionable variants with allele frequency ≥ 2% were retained.

For copy number variations detection, amplicons with read count in the lowest 5th percentile of all detectable amplicons and amplicons with a coefficient of variation ≥ 0.3 were removed. The remaining amplicons were normalized to correct the pool design bias. ONCOCNV [35] was applied to normalize total amplicon number, GC content, amplicon length, and technology-related biases, followed by segmenting the sample with a gene-aware model. The method was also used to establish the baseline of copy number variations from specimens in the ACT Genomics in-house database. Alterations are defined as putative mutation and CNV. The definition of putative mutations was based on the OncoPrinter from cBioPortal (https://www.cbioportal.org/). The mutations were classified into three catalogs, missense/in-frame (putative mutations without COSMIC ID), COSMIC ID (putative mutations with COSMIC ID), and nonsense/frameshift/splice-site. The CNVs were subdivided into three catalogs based on their changes. The amplification, gain, and deletion were defined as CN ≧ 8, 4 ≦ CN < 8, and CN = 0, respectively. All detected variants are listed in online resource Table S1.

### Tumor mutational burden calculation

Tumor mutational burden (TMB) was calculated using the sequenced regions of ACTOnco^®^, an in-house developed machine learning model with a cancer hotspot correction was applied to predict somatic variants and TMB calculation [36]. TMB results were shown as the number of somatic nonsynonymous mutations per megabase of all protein-coding genes (Mutations/Mb).

### Statistical analysis

We performed subgroup analyses to evaluate the associations between sexes and different clinical parameters. The chi-square test was used for comparing categorical outcomes when the group number is larger than or equal to 5, such as clinicopathology distribution by sex. Fisher’s exact test was used for comparing categorical outcomes when the subgroup is less than 5, the odds ratio, 95% confidence intervals (CI), and p-values of all enrichment analyses. We applied Benjamin-Hochberg correction for the number of independent tests conducted (significant using a False Discovery Rate of 0.05). The Mann–Whitney test was used for analyzing age and TMB distribution. The mutational signatures were performed using the MuSica website application [37]. The Kaplan–Meier survival curve, log-rank test, and Cox proportional hazard model were depicted for patients’ overall survival analyses. All the analysis was performed by Excel Office 2019 (Microsoft Inc., WA, USA), GraphPad Prism 9 (v. 9.2.0; GraphPad Inc., CA, USA), and R (v4.1.3). Tests are two-tailed tests, and *P* < 0.05 was considered statistically significant.

## Results

### Clinicopathological characteristics of the study population

The retrospective study consisted of 195 HCC patients, including 129 males (66.2%) and 66 females (33.8%; ratio of male to female = 1.95) patients. The clinicopathological characteristics of this cohort are summarized in Table 1. The median age at diagnosis was 62 (ranging from 26 to 86), and most patients were diagnosed in the early stage (Stage I and II, n = 150, 76.9%). Ninety-eight patients (50.3%) and 80 patients (41%) had HBV and HCV infection history, which is much higher than the TCGA non-Asian cohort (5.1% and 20.1%, respectively; Supplementary Table S1). The proportion of patients with cirrhosis, alcohol, and smoking history was 39%, 34.9%, and 46.2%, respectively.

**Table 1:** Clinical characteristics of 195 HCC patients enrolled in the study

Characteristics	Total patients (n = 195)	Male (n = 129)	Female (n = 66)	*P* value
Sex	–	66.2%	33.8%	–
Age				
Median (range)	62 (26–86)	62 (28–86)	61 (26–80)	0.7446^a^
Stage				
Early (I/II)	150 (76.9%)	96 (74.4%)	54 (81.8%)	0.2459^b^
Late (III/IV)	45 (23.1%)	33 (25.6%)	12 (18.2%)	
Viral infection				
HBV	98 (50.3%)	68 (52.7%)	30 (45.5%)	0.0867^b^
HCV	80 (41%)	48 (37.2%)	32 (48.5%)	
NBNC	17 (8.7%)	13 (10.1%)	4 (6.1%)	–
Cirrhosis				
Positive	76 (39%)	45 (34.9%)	31 (47%)	0.1015^b^
Negative	119 (61%)	84 (65.1%)	35 (53%)	
Alcohol				
Yes	68 (34.9%)	66 (51.2%)	2 (3%)	< 0.0001^b^
No	120 (61.5%)	60 (46.5%)	60 (90.9%)	
Unknown	7 (3.6%)	3 (2.3%)	4 (6.1%)	–
Smoking				
Yes	90 (46.2%)	86 (66.7%)	4 (6.1%)	< 0.0001^b^
No	97 (49.7%)	39 (30.2%)	58 (87.9%)	
Unknown	8 (4.1%)	4 (3.1%)	4 (6.1%)	–

*HBV* hepatitis B virus, *HCV* hepatitis C virus, *NBNC* non-B non-C

^a^Calculated by the Mann–Whitney test

^b^Calculated by chi-squared test

We further explored the associations between the clinicopathological features and patients’ sex. Compared with female patients, significantly more male patients have drinking and smoking habits (51.2% vs. 3% and 66.7% vs. 6.1%, respectively; both *P* values are smaller than 0.0001). There is no difference in the age at diagnosis, stage distribution, viral infection history, and cirrhosis distribution between male and female HCC patients.

### Mutational landscape in Taiwanese HCC

**Fig. 1:**
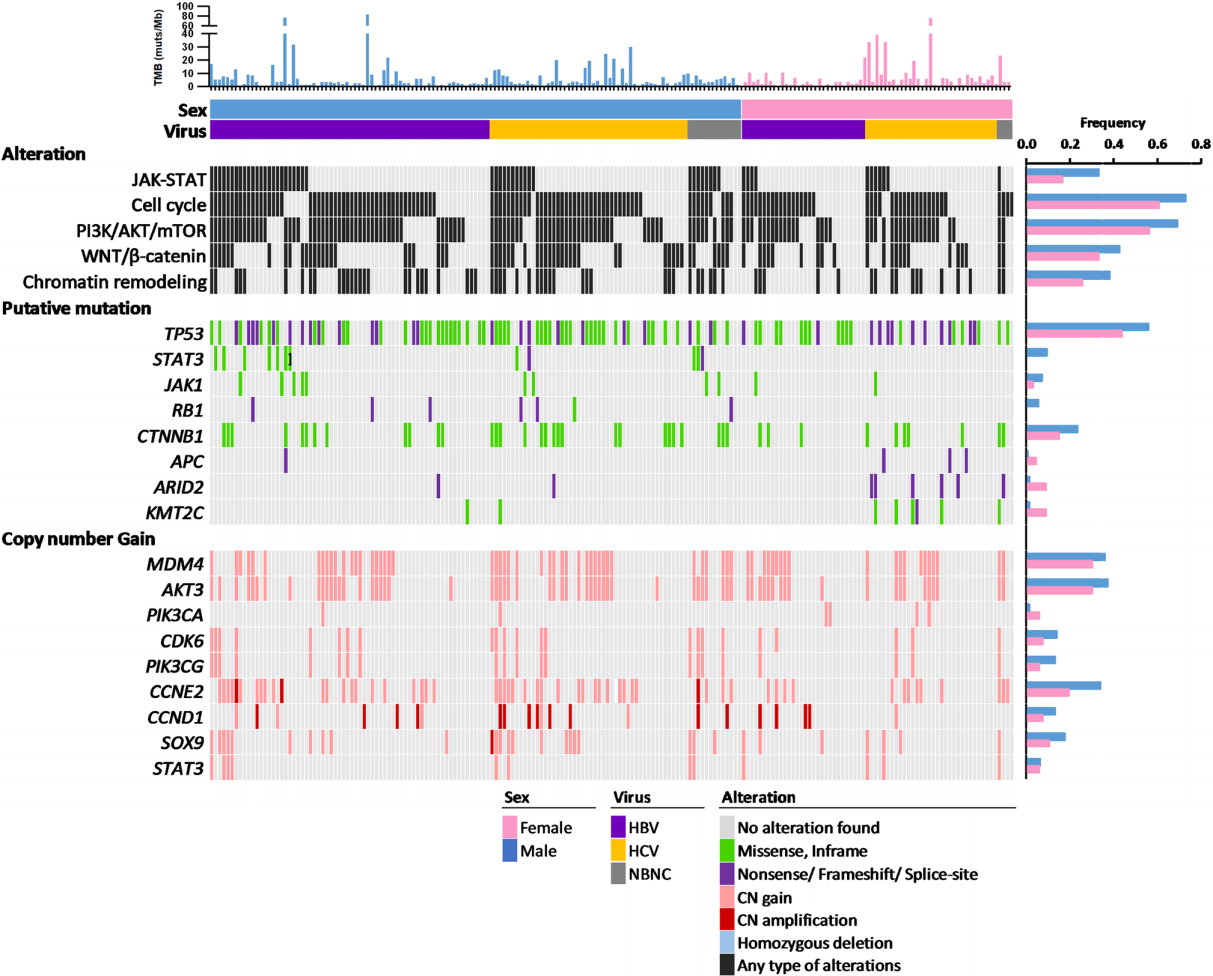
Mutational profiles of HCC specimens according to sex in the Taiwanese population. Each column represents one patient. Genes involved in the same signaling pathways are grouped, and the mutation frequencies among males and females are shown in the right plots. Each sample’s TMB is shown in the upper panel. Colors specify different types of alteration

The extracted genomic DNA was analyzed by targeted DNA sequencing on 440 cancer-related gene coding regions. Among the 195 specimens, we identified 6,156 alterations, including 1819 mutations and 4337 CNVs (listed in Supplementary Table S2 excel file). The most frequently altered genes and signaling pathways are illustrated in Fig. 1. As expected, *TP53* and *CTNNB1* are the top two genes most mutated in HCC [38–40]. In addition, we found that *ARID1A*, *ARID2*, *JAK1,* and *KMT2C* genes are recurrently mutated in the Taiwanese HCC specimen (as shown in Supplementary Fig S1).

Among the 4337 CNVs, 4215 (97.2%) were classified as copy number gain or amplification, and 122 (2.8%) were homozygous deletion. Our results confirmed the previously well-defined recurrent CNVs in HCC, including recurrent gains on chr 1q21.1-q23.3 (encoding genes like *BCL9*, *MCL1*, *NTRK1*, *DDR2*, *FCGR2B*, and *SDHC*), chr 1q31.1-q44 (encoding genes like *PTGS2*, *CDC73*, *IKBKE*, *BTG2*, *MDM4, PIK3C2B, USH2A, PARP1*, *FH*, and *AKT3*), chr 8q21.3-q24.3 (encoding genes like *NBN*, *RUNX1T1, CCNE2*, *UBR5*, *MYC*, and *RECQL4*), and chr 17q22-25.3 (encoding genes like *RNF43*, *BRIP1*, *CD79B*, *AXIN2*, *GNA13*, *PRKAR1A*, *SOX9*, and *RPTOR* (as shown in Supplementary Fig S1).

### Comparison of genomic profiles between genders in Taiwanese HCC patients

We compared the alteration frequencies of commonly mutated genes in male and female patient subgroups to elucidate the difference in genomic alteration patterns between male and female HCC patients in Taiwan (as shown in Fig. 1). Our analysis, presented in Supplementary Fig S1–2, revealed no significant difference in TMB between the sexes or with the presence of the virus. Notably, while a substantial disparity in smoking history was observed between male and female patients, no difference in TMB was found (*P* = 0.4653). We also cross-referenced our findings with the TCGA HCC dataset, and similar results were observed, with no significant variations in TMB between sexes, virus presence, or smoking history, as demonstrated in Supplementary Fig S3.

Figure 1 illustrates that the prevalence of *STAT3* mutations is higher in male patients compared to females (9.3% versus 0%, *P* = 0.0092). However, when we accounted for multiple comparisons, the difference did not maintain statistical significance (adjusted *P* = 0.135; Benjamini–Hochberg correction; Supplementary Table S4). We also investigated the mutation frequency of *CTNNB1*, a gene known for its sex-biased mutation pattern in liver hepatocellular carcinoma [24]. In the Taiwanese cohort, we observed mutation frequencies of *CTNNB1* in male and female HCC patients at 23.3% and 15.2%, respectively. However, these differences did not demonstrate statistical significance after adjustment (adjusted *P* = 0.520; Benjamini–Hochberg, Supplementary Table S4).

When analyzing copy number variations (CNVs) as depicted in Fig. 1 and Supplementary Fig S1, we found that more male patients harbored copy number gains of the 8q22.1 encoding the *CCNE2* gene, in comparison to females (34.9% vs. 19.7%; *P* = 0.038). However, this difference was no longer statistically significant after adjusting for multiple comparisons (adjusted *P* = 0.717; Benjamini–Hochberg; Supplementary Table S3). Regarding signal pathway analysis, our data indicated that male patients showed a higher likelihood of genomic alterations in the JAK–STAT (33.3% vs. 16.7%, *P* = 0.0139).

A previous study had reported an enrichment of sex-biased genes on the X chromosome [24]. In light of this, we sought to investigate whether there were differences in the mutational profiles of X-linked genes in HCC based on gender. A total of 20 X-linked genes were included in our panel, and the detailed analysis results are shown in Supplementary Table S4. Initially, our finding indicated that compared to male patients, more female patients with mutations in *BCOR*, *BTK*, and *XIAP* had *P* values of 0.0066, 0.0457, and 0.0376, respectively. However, when we applied a statistical adjustment for multiple comparisons to our data, the initial statistically significant difference we observed was no longer evident. Consequently, after applying stringent correction, we did not detect any statistically significant differences in the mutation patterns of these X-linked genes between males and females.

In terms of mutational signatures, we grouped and analyzed samples with a substantial number of mutations (≥ 5 mutations) based on sex and/or smoking status (Supplementary Table S5 and Fig S4). None of the female smokers met the criteria and were excluded from the analysis. Our findings revealed that the most prevalent mutational signature in the Taiwanese cohort was signature 22, which is associated with exposure to aristolochic acid. This was followed by signatures 1 (related to aging), 18 (possibly associated with damage by reactive oxygen species), 24 (associated with exposure to aflatoxin), 27 (of unknown etiology, possible sequencing artifact), and 28 (unknown etiology). As expected, the frequencies of mutation signatures associated with smoking (Signatures 4) and tobacco chewing (Signatures 29) were higher in males when compared to females. However, it is important to note that these differences did not reach statistical significance. Notably, the frequencies of signatures 15 (associated with defective DNA mismatch repair), 23 (unknown etiology), and 26 (related to defective DNA mismatch repair) were higher in smokers than non-smokers. In contrast, signature 27 (unknown etiology) was more prominent in non-smokers.

### Comparison of genomic profiles between genders in the TCGA HCC dataset

**Fig. 2:**
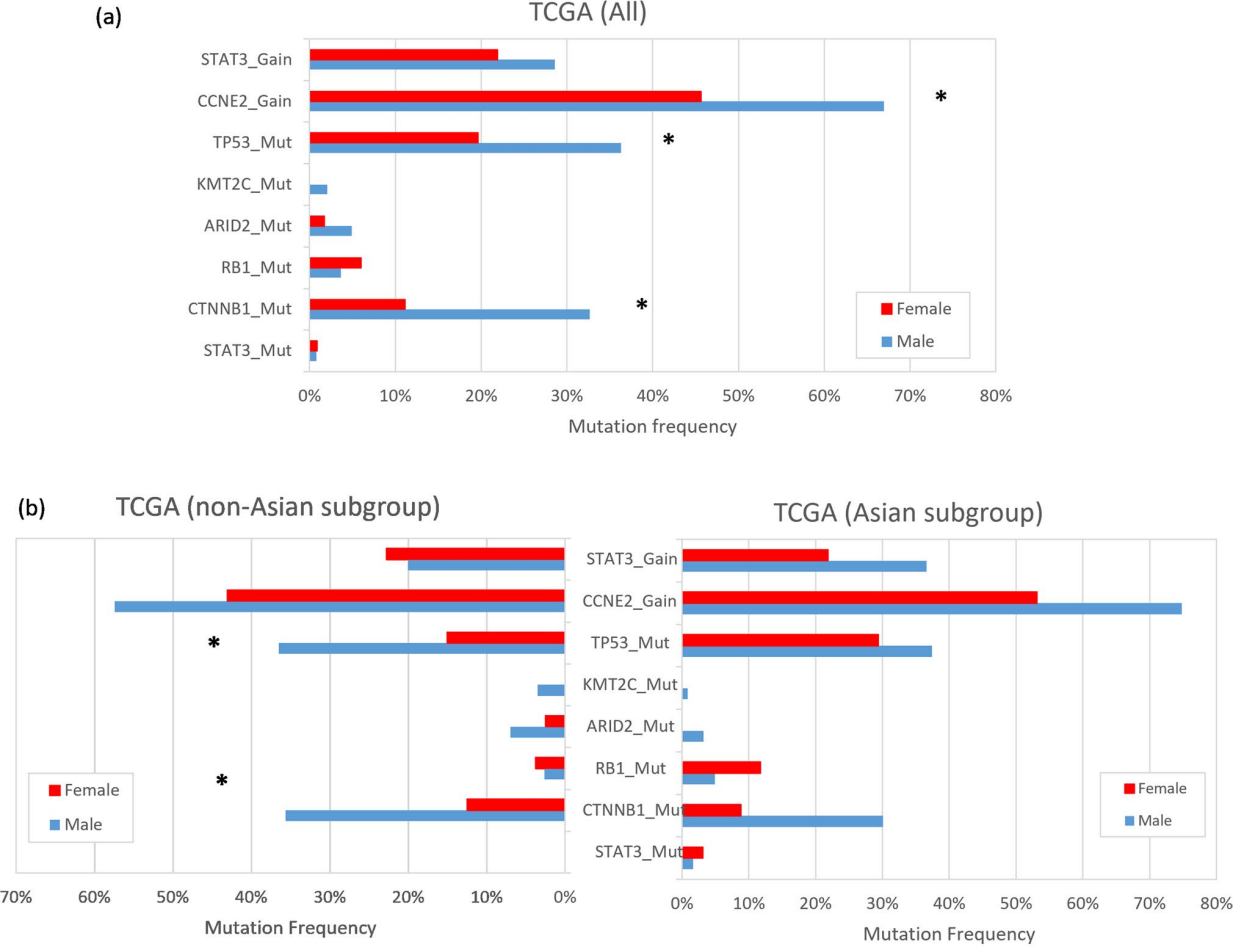
Alteration frequencies of driver genes by sex among the TCGA dataset. **a** All patients; **b** Asian and Non-Asian subgroups

Considering the limited sample size of the Taiwanese cohort, we extended our analysis to the TCGA HCC dataset to investigate the presence of sex-biased genomic alteration in subgroups stratified by ethnicity. In the TCGA dataset, we found that *CTNNB1*, *CCNE2*, and *TP53* were the genes that altered significantly more in males than in females (Fig. 2a and Supplementary Table S6). When we divided the dataset into Asian and non-Asian subgroups, the sex-biased mutation profile was only observed in the non-Asian subgroup. The mutation frequencies of *CTNNB1* and *TP53* were significantly higher in male patients than in females (35.65% vs. 12.5% and 36.52% vs. 15%, respectively; Fig. 2b), with adjusted *P* values of 0.0024 and 0.004. No significant gender-based differences in mutation profiles were found in the Asian subgroup.

In summary, our study did not identify sex-biased genomic alterations in the Taiwanese cohort. However, we observed higher mutation frequencies in the *CTNNB1* and *TP53* genes, and copy number gains in the *CCNE2* gene, in male patients in the TCGA dataset.

### Ethnic-specific genomic alterations

Meanwhile, we uncovered an interesting aspect regarding ethnic-specific genomic alterations. Specifically, we found several hotspot mutations in *JAK1* (S703I and S729C) and *STAT3* (Y640F) that may show an ethnic-specific distribution. In the Taiwanese cohort, 4 and 6 out of 195 (2.0% and 3.1%, respectively) carried *JAK1* p.S703I and p.S729C mutations, and 10 out of 195 (5.1%) patients harbored *STAT3* p.Y640F. However, when we examined the TCGA dataset, a notable difference emerged. In the Asian subgroup, *JAK1* p.S703I and p.S729C were identified in 1 (0.6%) and 2 (1.3%) out of 158 patients, but none were detected in the non-Asian subgroup (Supplementary Fig S5). Similarly, *STAT3* p.Y640F was found in 2 (1.2%) out of 158 Asian HCC patients but was absent in non-Asian patients (Supplementary Fig S6). Collectively, the results implied the presence of ethnic group-specific genomic alterations, although further research is needed to confirm these findings.

### Sex-specific prognostic markers

To investigate whether clinicopathological factors influence the prognosis of patients with HCC in the Taiwanese population, we retrieved survival data from the National Health Research Institutes Biobank database. The median overall survival (OS) was 80 months, with a range of 0.7 to 165 months. We compared OS based on various clinical parameters using Kaplan–Meier survival curves and log-rank tests. Supplementary Table S7 shows that our results revealed a significant difference in OS between patients with advanced-stage disease (stage III and IV) and those with early-stage disease (median OS = 35 months vs. 95 months, *P* = 0.0047; log-rank test). However, no significant impact on OS was found for age (*P* = 0.1011), viral infection (*P* = 0.5584), cirrhosis (*P* = 0.2274), alcohol consumption (*P* = 0.9018), and smoking (*P* = 0.7820). Additionally, there were no significant differences in OS between male and female patients (*P* = 0.7559), which aligns with previous observations [41].

**Fig. 3:**
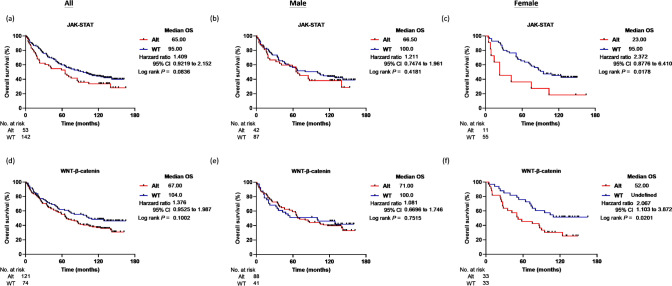
Kaplan–Meier survival analysis for HCC patients in the Taiwanese cohort. The overall survival (OS) was evaluated for all patients (**a**, **d**), male patients (**b**, **e**), and female patients (**c**, **f**) in the Taiwanese cohort. The analysis was based on the genomic status of alterations in the JAK–STAT and WNT-β-catenin pathways

Next, we explored the relationships between genomic alterations and overall survival within the Taiwanese population, specifically with respect to sex. Results are shown in Fig. 3**, **Supplementary S7, and detailed in Supplementary Table S8. We found that two male patients carrying *KMT2C*-mutated tumors had shorter OS than those with wild-type *KMT2C* (median OS: 12 and 80 months, respectively, *P* = 0.0034; Supplementary Fig. 7a–c). Additionally, a single male patient with a putative *APC* mutation exhibited an exceptionally short OS of 3 months, starkly contrasting patients with wild-type *APC* gene (median OS = 75.5 months, *P* < 0.0001; Supplementary Fig. 7d–f). Notably, in the TCGA Asian subgroup, we identified one male patient with a mutated *KMT2C* and another with a mutated *APC*; both had shorter OS compared to those with wild-type *KMT2C* or *APC* (Supplementary Fig. 8a–f). However, due to the limited number of patients carrying *KMT2C* and *APC* mutations, further research is warranted to draw definite conclusions.

Furthermore, we found that female patients with copy number gains of the *STAT3* gene (n = 4) tended to have a poorer prognosis compared to those with the wild-type (WT) *STAT3* gene (Supplementary Figure S7g–i and Table S8). The median OS of female patients with WT and *STAT3* gains were 92.5 months and 16 months, respectively. A similar trend was seen in the TCGA Asian subgroup, where female patients with *STAT3* gene copy gains (n = 7) had significantly shorter OS than females with WT *STAT3* gene (Supplementary Figure S8g–i). It is noteworthy that *STAT3* gain remained as an independent predictor of shorter OS after adjusting with the patient’s stage and age characteristics (Hazard ratio = 10.434, 95% CI 3.331–32.677, *P* < 0.001; Cox proportional hazard model; Table 2). This difference was not evident in the male subgroup or the non-Asian dataset. However, the limited number of *STAT3* alteration detected in the two studied cohorts suggests that these findings should be interpreted cautiously. Similar to the findings of *KMT2C* and *APC* mutations in male HCC patients, further validation in larger cohorts is essential to confirm their clinical significance.

**Table 2:** The results of Cox regression analysis

Variables	All			Male			Female		
	Hazard ratio^b^	95% CI	*P* value	Hazard ratio^b^	95% CI	*P* value	Hazard ratio^b^	95% CI	*P* value
*STAT3* Gain^a^	1.018	1.002–1.035	0.028	0.712	0.280–1.811	0.476	10.434	3.331–32.677	< 0.001
JAK/STAT Alt^a^	1.316	0.885–1.953	0.174	1.130	0.705–1.809	0.612	2.547	1.195–5.432	0.016
WNT Alt^a^	1.284	0.866–1.905	0.214	1.094	0.665–1.802	0.723	1.757	0.917–3.367	0.090

^a^Model includes predictor variables like patients’ age and stage characteristics

^b^Reference group: patients with non-altered or wild-type genes

**Fig. 4:**
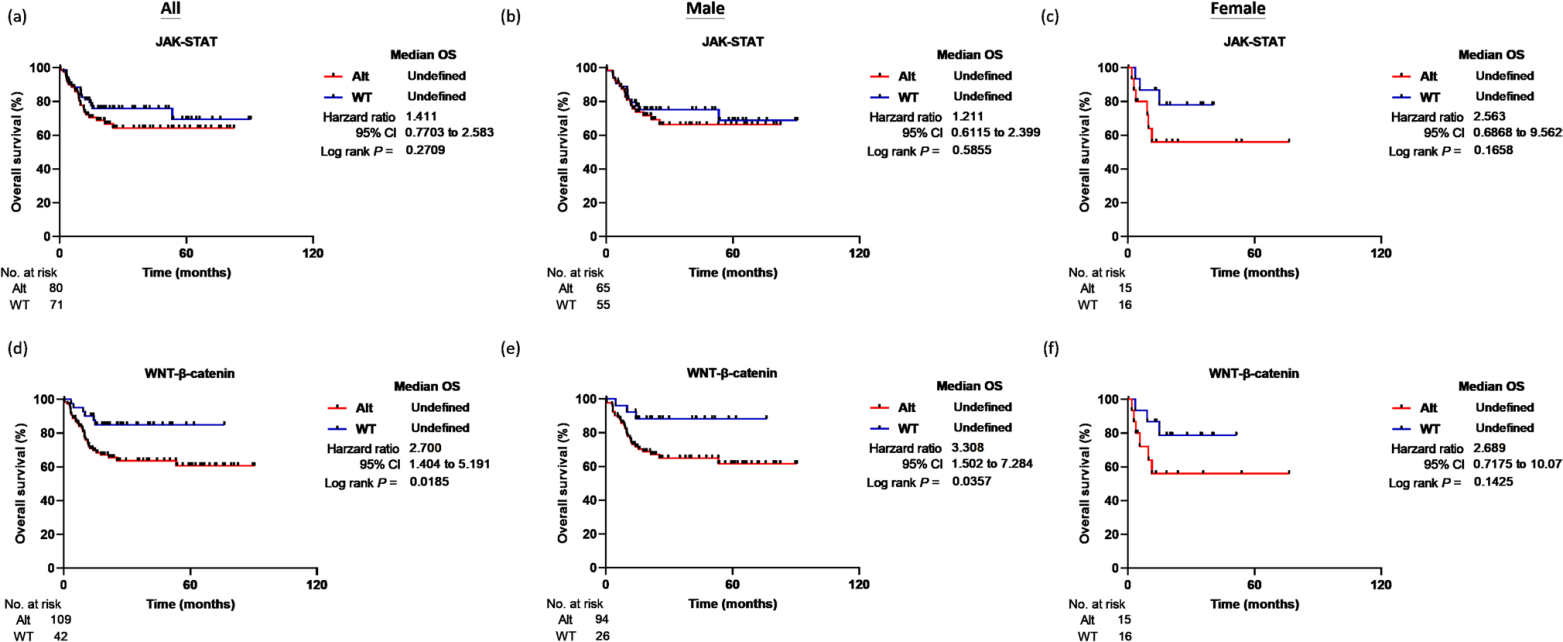
Kaplan–Meier survival analysis for HCC patients in the TCGA Asian subgroup. The overall survival (OS) was evaluated for all patients (**a**, **d**), male patients (**b**, **e**), and female patients (**c**, **f**). The analysis is based on the genomic status of alterations in the JAK–STAT and WNT-β-catenin pathways

From the pathway-based point of view, we observed female patients with altered JAK–STAT or Wnt-β-catenin pathways displayed a shorter overall survival than those with non-altered signal pathways, as shown in Fig. 3a–f. The median OS of female HCC patients with a non-altered JAK–STAT signaling pathway (n = 55) was 95 months, whereas patients with an altered JAK–STAT signaling pathway (n = 11) had a median OS of 23 months (*P* = 0.0178; log-rank test). After adjusting for patients’ stage and age, the alteration of the JAK–STAT pathway remained an independent factor associated with shorter OS (Hazard ratio = 2.547, 95% CI 1.195–5.432, *P* = 0.016; Cox proportional hazard model; Table 2). Furthermore, although not reaching statistical significance, a similar trend was observed in the TCGA Asian subgroup (n = 15, *P* = 0.1658; Fig. 4a–c). Female patients with an altered Wnt-β-catenin pathway also displayed poor OS (median OS = 52 months) compared to those with an unaltered Wnt-β-catenin pathway (*P* = 0.0201; log-rank test; Fig. 3d–f). However, in the TCGA Asian subgroup, a poor prognosis was observed in patients with altered Wnt-β-catenin pathway, irrespective of gender (Fig. 4d–f), yet, the results did not reach statistical significance after adjusting for patient age and stage characteristics (Table 2).

## Discussion

The substantial disparity in HCC incidence rates and aggressiveness between males and females is well-documented [42]. Here, we compared the genomic profiles using archived specimens from the Taiwanese population to evaluate whether sex-related genomic alteration contributes to this disparity. Within this specific cohort, we did not identify any sex-biased genomic alterations. However, we extended our investigation to the TCGA dataset and observed higher frequencies of gene copy gains in *CCNE2* and mutations in *CTNNB1* and *TP53* among male patients. Upon further stratification of the TCGA dataset by ethnicity, we found that the difference in mutation frequency of *CTNNB1* and *TP53* genes remained statistically significant within the non-Asian subgroup.

*TP53* is the most commonly mutated gene in human cancer, and it is noteworthy that *TP53* mutations are more prevalent in males than females among various non-reproductive solid tumors, including liver hepatocellular carcinoma [43]. Haupt et al. identified a cluster of genes located on the X chromosome that can influence the activity of the p53 protein. Males possessing only one X chromosome are particularly susceptible to developing diseases if genes on their X chromosome become dysfunctional. Furthermore, females display an exceptional incidence of non-expressed mutations among X-linked genes associated with p53. These intricate genetic dynamics may offer insights into the sex disparities observed in specific cancers [43]. In our study, we found that the mutation incidence of *TP53* in the Taiwanese population (51.8%) is markedly higher than that in the TCGA non-Asian cohort (27.6%; *P* < 0.00001; Chi-square test), which is consistent with previous study [44]. Collectively, these findings emphasize the importance of considering ethnic background and genetically-assigned sex potentially in the development of tailored therapeutic strategies.

The β-catenin protein encoded by *CTNNB1* is the major effector of WNT signaling. WNT signaling pathway plays a prominent role in the control of embryogenesis, stem cell renewal, cell fate determination, and cancer development [45, 46]. According to the TCGA data, activation of the WNT signaling pathway by *CTNNB1* or *AXIN1* mutation is found in a notable subset of HCC [46, 47]. Consistent with TCGA, *CTNNB1* is one of the top mutated genes in the Taiwanese HCC cohort and has a male-biased tendency [24], which predicts that males will receive greater therapeutic benefit from Wnt or β-catenin-targeting regimens. Therapeutic strategies targeting the WNT/b-catenin pathway have been studied for decades, including PORCN inhibitors, WNT ligand antagonists, FZD antagonists, and inhibitors of β-catenin transcriptional activity [48]. PRI-724 (ICG-001) is a selective inhibitor of the CREB-binding protein (CBP)/β-catenin. Preclinical studies have demonstrated the anti-tumor activity of PRI-724 in HCC cells with activated β-catenin [49]. Furthermore, a combination of ICG-001 and sorafenib showed a more significant growth-retarding effect in HCC cell lines [50]. Acceptable toxicity was observed in the phase I study of PRI-724 in patients with advanced solid tumors [NCT01302405]. However, only the anti-fibrotic effects of PRI-724, but not the anti-tumor effect, are currently evaluated in clinical trials [51]. Given the higher frequency of *CTNNB1* mutations in males among different ethnic groups, sex-related experimental design or clinical benefits of regimens targeting β-catenin should be considered in future studies.

Copy number gains of *CCNE2* were one of the three sex-biased genomic alterations that could be found in the TCGA dataset. *CCNE2* encodes cyclin E2 protein, a highly conserved cyclin family that regulates the function of cyclin-dependent kinase (CDK) function. Cyclin E2 interacts explicitly with and functions as a regulatory subunit of CDK2 and plays a crucial role in mitotic cell cycle regulation. *CCNE2* is located at chr 8q22.1, the region known to be recurrently altered in HCC and associated with high tumor grade and microvascular invasion [52]. However, unlike the cyclin E1 proteins, in vitro studies showed that cyclin E2 is not essential for initiating liver cancer [53]. Therefore, based on the current data, we cannot differentiate if gains of *CCNE2* or other genes located in this region 8q22.1-q24 are involved in the pathogenesis of HCC.

Our research has revealed two specific factors, copy number gain of *STAT3* and altered JAK–STAT pathway, as independent predictors of an unfavorable prognosis, particularly in female HCC patients. Remarkably, female patients with either a copy number gain of *STAT3* or alterations in the JAK–STAT pathway displayed significantly shorter OS than their male counterparts, with a median OS of 16 months and 23 months compared to 79 months and 66.5 months, respectively. The gender-based trend was a unique feature observed exclusively within the TCGA Asian subgroup and was absent in the non-Asian subgroup. Adding to this complexity, we identified two recurrent activating mutations in *JAK1* (S703I and S729C) and one in *STAT3* (Y640F), primarily observed in Taiwanese males. These mutations are well-known for their capability to trigger the constitutive phosphorylation and activation of STAT3 without external triggers in various cell lines [54, 55]. Importantly, these specific mutations were unique to the Asian population within the TCGA dataset.

These compelling findings underscore the potential significance of the JAK–STAT signaling pathway in the initiation or development of Asian HCC. At the same time, it is established that aberrant activation of the JAK–STAT signaling pathway and persistent activation of STAT3 is expected in approximately 60% of HCC cases, with a clear association with poor prognosis [56–58]. Our study, to the best of our knowledge, is the first to propose a gender-based impact of STAT3 copy number gains and JAK–STAT pathway on the prognosis of female HCC patients. Further investigation is warranted to explore this intriguing observation in more detail.

Furthermore, these results emphasize the urgent need for JAK–STAT targeting therapeutic approach for HCC, especially in the Asian population [56, 59]. Several STAT3 inhibitors have been advanced into clinical trials with promising therapeutic potential as monotherapy or in combination with other treatment modalities in HCC and other cancers. One notable example is Napabucasin (BBI608), a first-in-class cancer stemness inhibitor that suppresses cancer stemness by targeting STAT3-driven gene transcription [60]. A phase Ib/II clinical trial study in patients with HCC demonstrated encouraging anti-tumor activity and acceptable safety profile of napabucasin when combined with sorafenib [61].

We are fully aware that the major limitation of this study is the sample size (n = 195), which is relatively small, potentially limiting our ability to detect differences. Specifically, we were only able to identify the impact of sex on highly mutated genes or common copy number alterations. It is essential to acknowledge that variations in HCC mutational patterns are linked to diverse genetic backgrounds and environmental factors like smoking, alcohol consumption, exposure to aristolochic acid, and infections with HBV and HCV [62, 63]. While we sought to address these concerns by validating our initial findings with a more extensive dataset, the TCGA HCC dataset, our limited sample size still prevents us from determining whether the observed genomic distinctions between sexes arise from variations in etiology or ancestral population. Thirdly, the sequencing platform we used in our study is a targeted panel, which only contains the exons of 440 cancer-related genes. As genomic alterations led to sex disparity may not be limited to driver genes, a more comprehensive large-scale prospective study should be conducted to validate our findings.

HCC is a solid tumor showing a noticeable sex disparity, which occurs preferentially in males. Previous studies indicated that HCC in males and females are biologically distinct and may respond differently to treatments. In 2014, the US National Institutes of Health (NIH) developed policies encouraging investigate-initiated grants to make reasonable efforts to consider sex as a biological variable for medical research. However, no randomized controlled trials have demonstrated sex-related differences in HCC treatment response or clinical outcomes [64]. Considering the racial background and genetically assigned sex in clinical study designs and analyses is warranted in future trials to understand better whether and how these factors may modify treatment response to specific therapeutic targets and influence clinical outcomes.

## Conclusions

In the present study, we characterized the mutational landscapes among male and female patients in Taiwanese HCC. We pinpointed sex-specific molecular targets that may have implications for prognoses. These findings not only contribute to our understanding of sex disparity in HCC, particularly within the Asian population but also establish a foundation for future research in this area. The validation of our results through larger cohorts and extensive sequencing efforts is strongly recommended to advance our knowledge in this field further.

### Supplementary Information

Below is the link to the electronic supplementary material.

Supplementary file 1 (DOCX 1356 KB)

Supplementary file 2 (XLSX 328 KB)

## Data Availability

The sequencing raw data that support the findings of this study are available from the corresponding authors. However, certain restrictions apply to the availability of these data, which were used under license for the current study and are not publicly available. Data are however available from the authors upon reasonable request and with permission of the National Health Research Institutes.
