# Supplementary material for: Study of sex-biased differences in genomic profiles in East Asian hepatocellular carcinoma
Source: Discov Oncol. 2024 Jul 9;15:276. doi: 10.1007/s12672-024-01131-9 (PMC11233483; doi:10.1007/s12672-024-01131-9)

**Study of Sex-biased Differences in Genomic Profiles in East Asian Hepatocellular Carcinoma**

**Chung-Yu Huang^1^, Kien-Thiam Tan^1,2^, Shiu-Feng Huang^3^, Yen-Jung Lu^1^, Yeh-Han Wang^1,4,5^, Shu-Jen Chen^1*^, Ka-Po Tse^1*^**

^1^ ACT Genomics Co., Ltd., Taipei, Taiwan; [chloehuang@actgenomics.com](mailto:chloehuang@actgenomics.com) (C.-Y.H.); [jtchenbio@gmail.com](mailto:jtchenbio@gmail.com) (K.-T.T); [mimilu91@gmail.com](mailto:mimilu91@gmail.com) (Y.-J.L.); [yehanwang@gmail.com](mailto:yehanwang@gmail.com) (Y.-H.W.); [sjchen@actgenomics.com](mailto:sjchen@actgenomics.com) (S.-J.C.); [bobotse@actgenomics.com](mailto:bobotse@actgenomics.com) (K.-P.T.)

^2^ Anbogen Therapeutics, Inc., Taipei, Taiwan; [jtchenbio@gmail.com](mailto:jtchenbio@gmail.com) (K.-T.T.)

^3^ Institute of Molecular and Genomic Medicine, Core Pathology Lab, National Health Research Institutes, Miaoli, Taiwan; [sfhuang@nhri.edu.tw](mailto:sfhuang@nhri.edu.tw) (S.-F.H.)

^4^ Department of Pathology, Fu Jen Catholic University Hospital, Fu Jen Catholic University, New Taipei City, Taiwan; [yehanwang@gmail.com](mailto:yehanwang@gmail.com) (Y.-H.W.)

^5^ School of Medicine, College of Medicine, Fu Jen Catholic University, New Taipei City, Taiwan; [yehanwang@gmail.com](mailto:yehanwang@gmail.com) (Y.-H.W.)

*Correspondence: Shu-Jen Chen: [sjchen@actgenomics.com](mailto:sjchen@actgenomics.com) and Ka-Po Tse: [bobotse@actgenomics.com](mailto:bobotse@actgenomics.com)

**Supplementary Materials:**

Table S1 Clinical characteristics of 356 HCC patients in the TCGA dataset

Table S2 Variants list from 195 Taiwanese HCC patients (Excel file)

Table S3 Frequencies of genomics alterations in the Taiwanese cohort by sex

Table S4 Mutation frequencies of X-linked genes in the Taiwanese cohort

Table S5 Frequencies of mutational signatures in the Taiwanese cohort

Table S6 Frequencies of genomic alterations in the TCGA stratified by ethnicity and sex

Table S7 Univariate test of overall survival in 195 Taiwanese HCC patients stratified by sex

Table S8 Univariate test of overall survival in 195 Taiwanese patients with HCC by sex

Fig S1 Mutational landscape of HCC patients in the Taiwanese population

Fig S2 Tumor mutation burden (TMB) in male and female HCC patients in the Taiwan population, stratified by hepatitis infection history and smoking status

Fig S3 Tumor mutation burden (TMB) in male and female HCC patients in the TCGA dataset, stratified by hepatitis infection history and smoking status

Fig S4 Distribution of mutational signatures in our studied cohort

Fig S5 Lollipop plots illustrate the distribution and mutation profiles of the *JAK1* gene in different cohorts

Fig S6 Lollipop plots illustrate the distribution and mutation profiles of the *STAT3* gene in different cohorts

**Table S1 Clinical characteristics of 356 HCC patients in the TCGA dataset**

| TCGA | Non-Asian (n = 198) | | | | Asian (n = 158) | | | |
| --- | --- | --- | --- | --- | --- | --- | --- | --- |
| Characteristics | All | Male | Female | *P* value | All | Male | Female | *P* value |
| Sex | 198 | 117 (59.1) | 81 (40.9) | - | 158 | 124 (78.5%) | 34 (21.5%) | - |
| Age |  |  |  |  |  |  |  |  |
| Median (range) | 65.5 (16-85) | 65 (16-81) | 66 (17-85) | 0.8173 ^a^ | 61 (18-76) | 55 (18-76) | 59 (20-74) | 0.4128 ^a^ |
| Stage |  |  |  |  |  |  |  |  |
| Early (I/II) | 131 (66.2) | 83 (70.9) | 48 (59.3) | 0.0812 ^b^ | 115 (72.8%) | 91 (73.4%) | 24 (70.6%) | 0.6626^b^ |
| Late (III/IV) | 46 (23.2) | 22 (18.8) | 24 (29.6) |  | 41(25.9%) | 31 (25%) | 10 (29.4%) |  |
| Unknown | 21 (10.6) | 12 (10.3) | 9 (11.1) | - | 2 (1.3%) | 2 (1.6%) | 0 | - |
| Viral infection |  |  |  |  |  |  |  |  |
| HBV | 10 (5.1) | 7 (6.0) | 3 (3.7) | 0.6690 ^b^ | 87 (55.1%) | 73 (58.9%) | 14 (41.2%) | >0.9999^b^ |
| HCV | 41 (20.7) | 33 (28.2) | 8 (9.9) |  | 6 (3.8%) | 5 (4%) | 1 (2.9%) |  |
| HBV+HCV | 4 (2.0) | 4 (3.4) | 0 | - | 48 (30.4%) | 46 (37.1%) | 2 (5.9%) | - |
| NBNC | 143 (72.2) | 73 (62.4) | 70 (86.4) | - | 17 (10.7%) | 0 | 17 (50%) | - |
| Cirrhosis |  |  |  |  |  |  |  |  |
| Positive | 35 (17.7) | 24 (20.5) | 11 (13.6) | 0.1109 ^b^ | 39 (24.7%) | 32 (25.8%) | 7 (20.6%) | 0.3334^b^ |
| Negative | 95 (48.0) | 49 (41.9) | 46 (56.8) |  | 44 (27.8%) | 40 (32.3%) | 4 (11.8%) |  |
| Unknown | 68 (34.3) | 44 (37.6) | 24 (29.6) | - | 75 (47.5%) | 52 (41.9%) | 3 (67.6%) | - |
| Alcohol |  |  |  |  |  |  |  |  |
| Yes | 73 (36.9) | 59 (50.4) | 14 (17.2) | < 0.0001 ^b^ | 43 (27.2%) | 43 (34.7%) | 0 | <0.0001^b^ |
| No | 125 (63.1) | 58 (49.6) | 67 (82.7) |  | 115 (72.8%) | 81 (65.3%) | 34 (100%) |  |
| Smoking |  |  |  |  |  |  |  |  |
| Yes | 10 (5.1) | 7 (6.0) | 3 (3.7) | 0.5255 ^b^ | 2 (1.3%) | 2 (1.6%) | 0 | >0.9999^b^ |
| No | 184 (92.9) | 106 (90.6) | 78 (96.3) |  | 149 (94.3%) | 115 (92.7%) | 34 (100%) |  |
| Unknown | 4 (2.0) | 4 (3.4) | 0 | - | 7 (4.4%) | 7 (5.7%) | 0 | - |

Abbreviations: HBV, hepatitis B virus. HCV hepatitis C virus. NBNC, non-B non-C.

^a^Calculated by the Mann-Whitney test.

^b^Calculated by chi-squared test.

**Table S3 Frequencies of genomics alterations in the Taiwanese cohort by sex**

| Variants | Frequency in Male | Frequency in Female | *P* values | Benjamini-Hochberg Adjusted *P* value | Significant using an FDR of 0.05 |
| --- | --- | --- | --- | --- | --- |
| Mutated *STAT3* | 9.3% | 0% | 0.033 | 0.135 | No |
| Mutated *CTNNB1* | 23.3% | 15.2% | 0.185 | 0.520 | No |
| Mutated *RB1* | 5.4% | 0% | 0.097 | 0.404 | No |
| Mutated *KMT2C* | 1.6% | 9.1% | 0.031 | 0.135 | No |
| Mutated *ARID2* | 1.6% | 9.1% | 0.031 | 0.135 | No |
| Mutated *APC* | 0.8% | 4.5% | 0.241 | 0.507 | No |
| *CCNE2* Gains | 34.9% | 19.7% | 0.038 | 0.717 | No |

Abbreviations: *STAT3*, Signal Transducer And Activator Of Transcription 3; *CTNNB1*, Catenin Beta 1; *KMT2C*, Lysine Methyltransferase 2C; *ARID2*, AT-Rich Interaction Domain 2; *APC*, APC Regulator Of WNT Signaling Pathway; *CCNE2*, Cyclin E2; *MDM4*, MDM4 Regulator Of P53; *AKT3*, AKT Serine/Threonine Kinase 3; *PIK3CG*, Phosphatidylinositol-4,5-Bisphosphate 3-Kinase Catalytic Subunit Gamma; *SOX9*, SRY-Box Transcription Factor 9; *PIK3CA*, Phosphatidylinositol-4,5-Bisphosphate 3-Kinase Catalytic Subunit Alpha; FDR, False Discovery Rate.

**Table S4 Mutation frequencies of X-linked genes in the Taiwanese cohort**

|  | Female | | |  | Male | | | P-value | Adjusted p-value^a^ |
| --- | --- | --- | --- | --- | --- | --- | --- | --- | --- |
| Genes | WT | Mut | Mut (%) |  | WT | Mut | Mut (%) |  |  |
| *AMER1* | 65 | 1 | 1.52% |  | 127 | 2 | 1.55% | 0.1710 | 0.3420 |
| *AR* | 64 | 2 | 3.03% |  | 127 | 2 | 1.55% | 0.6051 | 0.8378 |
| *ARAF* | 66 | 0 | 0.00% |  | 129 | 0 | 0.00% | 1.0000 | 1.0000 |
| *ATRX* | 63 | 3 | 4.55% |  | 126 | 3 | 2.33% | 0.4087 | 0.6131 |
| *BCOR* | 60 | 6 | 9.09% |  | 128 | 1 | 0.78% | 0.0066 | 0.1107 |
| *BTK* | 62 | 4 | 6.06% |  | 128 | 1 | 0.78% | 0.0457 | 0.1645 |
| *CCNB3* | 61 | 5 | 7.58% |  | 125 | 4 | 3.10% | 0.1695 | 0.3420 |
| *CRLF2* | 65 | 1 | 1.52% |  | 125 | 4 | 3.10% | 0.6640 | 0.8537 |
| *GATA1* | 65 | 1 | 1.52% |  | 128 | 1 | 0.78% | 1.0000 | 1.0000 |
| *KDM5C* | 63 | 3 | 4.55% |  | 129 | 0 | 0.00% | 0.0376 | 0.1645 |
| *KDM6A* | 65 | 1 | 1.52% |  | 127 | 2 | 1.55% | 1.0000 | 1.0000 |
| *MED12* | 63 | 3 | 4.55% |  | 128 | 1 | 0.78% | 0.1134 | 0.2916 |
| *PAK3* | 65 | 1 | 1.52% |  | 129 | 0 | 0.00% | 0.3385 | 0.5539 |
| *RBM10* | 63 | 3 | 4.55% |  | 128 | 1 | 0.78% | 0.1134 | 0.2916 |
| *SH2D1A* | 66 | 0 | 0.00% |  | 128 | 1 | 0.78% | 1.0000 | 1.0000 |
| *STAG2* | 63 | 3 | 4.55% |  | 127 | 2 | 1.55% | 0.3383 | 0.5539 |
| *TAF1* | 62 | 4 | 6.06% |  | 129 | 0 | 0.00% | 0.0123 | 0.1107 |
| *XIAP* | 63 | 3 | 4.55% |  | 129 | 0 | 0.00% | 0.0376 | 0.1645 |

^a^Adjusted by Benjamin-Hochberg correction.

**Table S5 Frequencies of mutational signatures in the Taiwanese cohort**

| **Signatures** | **All (n=117)** | **All** | | | | |  |  | **Male** | |  |  | **Female** |
| --- | --- | --- | --- | --- | --- | --- | --- | --- | --- | --- | --- | --- | --- |
|  |  | **Male (n=78)** | **Female (n=39)** | ***P* value^a^** | **Smoker (n=52)** | **Non-smoker (n=65)** | ***P* value^a^** |  | **Smoker (n=52)** | **Non-smoker (n=26)** | ***P* value^a^** |  | **Non-smoker (n=39)** |
| 1 | 28.2% | 24.4% | 35.9% | 0.1911 | 25.0% | 30.8% | 0.4908 |  | 25.0% | 23.1% | 0.8520 |  | 35.9% |
| 2 | 12.8% | 12.8% | 12.8% | >0.9999 | 9.6% | 15.4% | 0.3537 |  | 9.6% | 19.2% | 0.2311 |  | 12.8% |
| 3 | 8.5% | 7.7% | 10.3% | 0.7293 | 3.8% | 12.3% | 0.1816 |  | 3.8% | 15.4% | 0.0914 |  | 10.3% |
| 4 | 14.5% | 17.9% | 7.7% | 0.1714 | 19.2% | 10.8% | 0.1969 |  | 19.2% | 15.4% | 0.7636 |  | 7.7% |
| 5 | 1.7% | 2.6% | 0.0% | 0.5517 | 3.8% | 0.0% | 0.1954 |  | 3.8% | 0.0% | 0.5498 |  | 0.0% |
| 6 | 14.5% | 14.1% | 15.4% | 0.8528 | 15.4% | 13.8% | 0.8145 |  | 15.4% | 11.5% | 0.7429 |  | 15.4% |
| 7 | 15.4% | 11.5% | 23.1% | 0.103 | 11.5% | 18.5% | 0.3024 |  | 11.5% | 11.5% | >0.9999 |  | 23.1% |
| 8 | 15.4% | 16.7% | 12.8% | 0.5867 | 17.3% | 13.8% | 0.6061 |  | 17.3% | 15.4% | >0.9999 |  | 12.8% |
| 9 | 6.8% | 6.4% | 7.7% | >0.9999 | 3.8% | 9.2% | 0.2971 |  | 3.8% | 11.5% | 0.3264 |  | 7.7% |
| 10 | 16.2% | 14.1% | 20.5% | 0.3755 | 11.5% | 20.0% | 0.2175 |  | 11.5% | 19.2% | 0.3575 |  | 20.5% |
| 11 | 9.4% | 9.0% | 10.3% | >0.9999 | 9.6% | 9.2% | 0.9435 |  | 9.6% | 7.7% | >0.9999 |  | 10.3% |
| 12 | 13.7% | 14.1% | 12.8% | 0.8491 | 13.5% | 13.8% | 0.9520 |  | 13.5% | 15.4% | >0.9999 |  | 12.8% |
| 13 | 17.1% | 21.8% | 7.7% | 0.0697 | 21.2% | 13.8% | 0.2968 |  | 21.2% | 23.1% | 0.8462 |  | 7.7% |
| 14 | 9.4% | 6.4% | 15.4% | 0.1169 | 5.8% | 12.3% | 0.3415 |  | 5.8% | 7.7% | >0.9999 |  | 15.4% |
| 15 | 14.5% | 16.7% | 10.3% | 0.4169 | 23.1% | 7.7% | **0.0190** |  | 23.1% | 3.8% | 0.0500 |  | 10.3% |
| 16 | 13.7% | 15.4% | 10.3% | 0.5736 | 19.2% | 9.2% | 0.1177 |  | 19.2% | 7.7% | 0.3181 |  | 10.3% |
| 17 | 17.1% | 15.4% | 20.5% | 0.4873 | 19.2% | 15.4% | 0.5829 |  | 19.2% | 7.7% | 0.3181 |  | 20.5% |
| 18 | 22.2% | 23.1% | 20.5% | 0.7532 | 21.2% | 23.1% | 0.8037 |  | 21.2% | 26.9% | 0.5686 |  | 20.5% |
| 19 | 8.5% | 10.3% | 5.1% | 0.4925 | 11.5% | 6.2% | 0.3361 |  | 11.5% | 7.7% | 0.7118 |  | 5.1% |
| 20 | 14.5% | 14.1% | 15.4% | 0.8528 | 11.5% | 16.9% | 0.4115 |  | 11.5% | 19.2% | 0.3575 |  | 15.4% |
| 21 | 17.9% | 19.2% | 15.4% | 0.6093 | 13.5% | 21.5% | 0.2580 |  | 13.5% | 30.8% | 0.0675 |  | 15.4% |
| 22 | 82.1% | 79.5% | 87.2% | 0.3067 | 80.8% | 83.1% | 0.7465 |  | 80.8% | 76.9% | 0.6917 |  | 87.2% |
| 23 | 15.4% | 17.9% | 10.3% | 0.4156 | 23.1% | 9.2% | **0.0391** |  | 23.1% | 7.7% | 0.1240 |  | 10.3% |
| 24 | 23.9% | 25.6% | 20.5% | 0.54 | 23.1% | 24.6% | 0.8463 |  | 23.1% | 30.8% | 0.4633 |  | 20.5% |
| 25 | 14.5% | 17.9% | 7.7% | 0.1714 | 21.2% | 9.2% | 0.0690 |  | 21.2% | 11.5% | 0.3633 |  | 7.7% |
| 26 | 3.4% | 5.1% | 0.0% | 0.2997 | 7.7% | 0.0% | **0.0365** |  | 7.7% | 0.0% | 0.2951 |  | 0.0% |
| 27 | 26.5% | 23.1% | 33.3% | 0.236 | 17.3% | 33.8% | **0.0440** |  | 17.3% | 34.6% | 0.0872 |  | 33.3% |
| 28 | 22.2% | 23.1% | 20.5% | 0.7532 | 26.9% | 18.5% | 0.2740 |  | 26.9% | 15.4% | 0.3930 |  | 20.5% |
| 29 | 14.5% | 15.4% | 12.8% | 0.7106 | 15.4% | 13.8% | 0.8145 |  | 15.4% | 15.4% | >0.9999 |  | 12.8% |
| 30 | 10.3% | 10.3% | 10.3% | >0.9999 | 5.8% | 13.8% | 0.2220 |  | 5.8% | 19.2% | 0.1086 |  | 10.3% |

^a^Calculated by chi-square test.

**Table S6** **Frequencies of genomic alterations in the TCGA stratified by ethnicity and sex**

| **Gene** | **Types of alteration** | **All** | | | |  | **Asian subgroup** | | | |  | **Non-Asian subgroup** | | | |
| --- | --- | --- | --- | --- | --- | --- | --- | --- | --- | --- | --- | --- | --- | --- | --- |
|  |  | **Male** | **Female** | **P value^a^** | **Adjusted P value^b^** |  | **Male** | **Female** | **P value^a^** | **Adjusted P value^b^** |  | **Male** | **Female** | **P value^a^** | **Adjusted P value^b^** |
| *STAT3* | Mut | 0.82% | 0.85% | >0.9999 | >0.9999 |  | 1.64% | 3.13% | 0.5054 | 0.6643 |  | 0.00% | 0.00% | >0.999 | >0.999 |
| *CTNNB1* | Mut | 32.65% | 11.11% | <0.0001 | 0.0004 |  | 30.08% | 8.82% | 0.0134 | 0.0672 |  | 35.65% | 12.50% | 0.0003 | 0.0024 |
| *RB1* | Mut | 3.67% | 5.98% | 0.3174 | 0.3627 |  | 4.88% | 11.76% | 0.2251 | 0.4502 |  | 2.61% | 3.75% | 0.6908 | 0.7894 |
| *ARID2* | Mut | 4.90% | 1.71% | 0.2417 | 0.2947 |  | 3.25% | 0.00% | 0.5813 | 0.6643 |  | 6.96% | 2.50% | 0.2025 | 0.3240 |
| *KMT2C* | Mut | 2.04% | 0% | 0.1795 | 0.2947 |  | 0.82% | 0.00% | >0.999 | >0.999 |  | 3.48% | 0.00% | 0.1454 | 0.2908 |
| *TP53* | Mut | 36.33% | 19.66% | 0.0013 | 0.0035 |  | 37.40% | 29.41% | 0.3895 | 0.6232 |  | 36.52% | 15.00% | 0.0010 | 0.004 |
| *CCNE2* | Gain | 66.94% | 45.61% | 0.0001 | 0.0004 |  | 74.80% | 53.13% | 0.0168 | 0.0672 |  | 57.39% | 43.04% | 0.0494 | 0.1317 |
| *STAT3* | Gain | 28.57% | 21.93% | 0.1842 | 0.2947 |  | 36.59% | 21.88% | 0.1164 | 0.0672 |  | 20.00% | 22.78% | 0.6407 | 0.7894 |

Abbreviations: Mut, mutation; Gain, copy number gain.

^a^Calculated by Fisher’s exact test.

^b^Adjusted by Benjamin-Hochberg correction.

**Table S7** **Univariate test of overall survival in 195 Taiwanese HCC patients stratified by sex**

| Characteristics | All patients (n=195) | | Male (n = 129) |  | Female (n = 66) |  |
| --- | --- | --- | --- | --- | --- | --- |
|  | HR (95% CI) | *P* value | HR (95% CI) | *P* value | HR (95% CI) | *P* value |
| Age |  |  |  |  |  |  |
| ≧65 | 1.35 (0.9340-1.9500) | 0.1011 | 1.372 (0.8739-2.1550) | 0.1579 | 1.309 (0.6925-2.4760) | 0.3923 |
| < 65 | Ref. |  | Ref. |  | Ref. |  |
| Sex |  |  |  |  |  |  |
| Female | Ref. | 0.7559 | Ref. | - | Ref. | - |
| Male | 1.062 (0.7280-1.5490) |  | Ref. |  | Ref. |  |
| Stage |  |  |  |  |  |  |
| Early (I/II) | Ref. | 0.0047 | Ref. | 0.1102 | Ref. | 0.0020 |
| Late (III/IV) | 1.766 (1.1050-2.8250) |  | 1.479 (0.8658-2.5260) |  | 2.897 (1.0460-8.0230) |  |
| Viral infection |  |  |  |  |  |  |
| HBV | 0.8935 (0.6109-1.3070) | 0.5584 | 0.8926 (0.5535-1.4390) | 0.6366 | 0.8823 (0.4669-1.6670) | 0.6985 |
| HCV | Ref. |  | Ref. |  | Ref. |  |
| NBNC | Ref. |  | Ref. |  | Ref. |  |
| Cirrhosis |  |  |  |  |  |  |
| Positive | 1.249 (0.8624-1.8090) | 0.2274 | 1.193 (0.7479-1.904) | 0.4445 | 1.411 (0.7579-2.6260) | 0.2735 |
| Negative | Ref. |  | Ref. |  | Ref. |  |
| Alcohol |  |  |  |  |  |  |
| Yes | 0.9762 (0.6654-1.4320) | 0.9018 | 1.035 (0.600-1.6230) | 0.8801 | Ref. | 0.1520 |
| No | Ref. |  | Ref. |  | Ref. |  |
| Unknown | Ref. | - | Ref. | - | Ref. | - |
| Smoking |  |  |  |  |  |  |
| Yes | 1.053 (0.7278-1.5240) | 0.7820 | 1.099 (0.6784-1.7800) | 0.7047 | 0.7432 (0.2130-2.5940) | 0.6800 |
| No | Ref. |  | Ref. |  | Ref. |  |
| Unknown | Ref. | - | Ref. | - | Ref. | - |

Abbreviations: HR, hazard ratio; C.I., confidence interval; Ref., reference; HBV, hepatitis B virus. HCV hepatitis C virus. NBNC, non-B non-C.

**Table S8** **Univariate test of overall survival in 195 Taiwanese patients with HCC by sex**

| Characteristics | Factor | All (n = 195) | |  | Male (n = 129) | | |  | Female (n = 66) | | |
| --- | --- | --- | --- | --- | --- | --- | --- | --- | --- | --- | --- |
|  |  | No. of patients (%) | Log-rank |  | No. of patients (%) | Log-rank | |  | No. of patients (%) | Log-rank | |
|  |  |  | p-value |  |  | Medium OS (months) | p-value |  |  | Medium OS (months) | p-value |
| Stage | Early | 150 (76.9) | 0.0047 |  | 96 (74.4) | - | 0.11 |  | 54 (81.8) | 25 | 0.002 |
|  | Late | 45 (23.1) |  |  | 33 (25.6) |  |  |  | 12 (18.2) | 99.5 |  |
| Cell cycle | Alt | 134 (68.7) | 0.4773 |  | 94 (72.9) | - | 0.9513 |  | 40 (60.6) | - | 0.2520 |
|  | WT | 61 (31.3) |  |  | 35 (27.1) |  |  |  | 26 (39.4) |  |  |
| *CCNE2* | Gain | 58 (29.7) | 0.1433 |  | 45 (34.9) | - | 0.3530 |  | 13 (19.7) | - | 0.2436 |
|  | WT | 137 (70.3) |  |  | 84 (65.1) |  |  |  | 53 (80.3) |  |  |
| *CCND1* | Gain | 22 (11.3) | 0.2406 |  | 17 (13.2) | - | 0.1686 |  | 5 (7.6) | - | 0.8203 |
|  | WT | 173 (88.7) |  |  | 112 (86.8) |  |  |  | 61 (92.4) |  |  |
| *RB1* | Mut | 7 (3.6) | 0.8696 |  | 7 (5.4) | - | 0.8248 |  | 0 | - | - |
|  | WT | 188 (96.4) |  |  | 122 (94.6) |  |  |  | 66 (100.0) |  |  |
| *MDM4* | Gain | 66 (33.8) | 0.7037 |  | 46 (35.7) | - | 0.3146 |  | 20 (30.3) | - | 0.4020 |
|  | WT | 129 (66.2) |  |  | 83 (64.3) |  |  |  | 46 (69.7) |  |  |
| *CDK6* | Gain | 23 (11.8) | 0.1995 |  | 18 (14.0) | - | 0.3462 |  | 5 (7.6) | - | 0.3575 |
|  | WT | 172 (88.2) |  |  | 111 (86.0) |  |  |  | 61 (92.4) |  |  |
| PI3K-Akt-mTOR | Alt | 126 (64.6) | 0.9056 |  | 89 (69.0) | - | 0.1802 |  | 37 (56.1) | - | 0.0636 |
|  | WT | 69 (35.4) |  |  | 40 (31.0) |  |  |  | 29 (43.9) |  |  |
| *AKT3* | Gain | 68 (34.9) | 0.8873 |  | 48 (37.2) | - | 0.9905 |  | 20 (30.3) | - | 0.861 |
|  | WT | 127 (65.1) |  |  | 81 (62.8) |  |  |  | 46 (69.7) |  |  |
| *PIK3CG* | Gain | 21 (10.8) | 0.3701 |  | 17 (13.2) | - | 0.4967 |  | 4 (6.1) | - | 0.5528 |
|  | WT | 174 (89.2) |  |  | 112 (86.8) |  |  |  | 62 (93.9) |  |  |
| *PI3KCA* | Gain | 6 (3.1) | 0.1022 |  | 2 (1.6) | - | 0.0974 |  | 4 (6.1) | - | 0.3399 |
|  | WT | 189 (96.9) |  |  | 127 (98.4) |  |  |  | 62 (93.9) |  |  |
| *RPTOR* | Gain | 48 (24.6) | 0.5497 |  | 35 (27.1) | - | 0.1818 |  | 13 (19.7) | - | 0.3008 |
|  | WT | 147 (75.4) |  |  | 94 (72.9) |  |  |  | 53 (80.3) |  |  |
| *AKT2* | Gain | 12 (6.2) | 0.7147 |  | 7 (5.4) | - | 0.9722 |  | 5 (7.6) | - | 0.5427 |
|  | WT | 183 (93.8) |  |  | 122 (94.6) |  |  |  | 61 (92.4) |  |  |
| WNT-β-catenin | Alt | 121 (62.1) | 0.1002 |  | 88 (68.2) | - | 0.7515 |  | 33 (50) | - | 0.0201 |
|  | WT | 74 (37.9) |  |  | 41 (31.8) |  |  |  | 33 (50) |  |  |
| *CTNNB1* | Mut | 40 (20.5) | 0.0735 |  | 30 (23.3) | - | 0.2361 |  | 10 (15.2) | - | 0.1566 |
|  | WT | 155 (79.5) |  |  | 99 (76.7) |  |  |  | 56 (84.8) |  |  |
| *APC* | Mut | 4 (2.1) | 0.0412 |  | 1 (0.8) | 3 | <0.0001 |  | 3 (4.5) | - | 0.1583 |
|  | WT | 191 (97.9) |  |  | 128 (99.2) | 75.5 |  |  | 63 (95.5) |  |  |
| *SOX9* | Gain | 30 (15.4) | 0.9222 |  | 23 (17.8) | - | 0.5507 |  | 7 (10.6) | - | 0.3646 |
|  | WT | 165 (84.6) |  |  | 106 (82.2) |  |  |  | 59 (89.4) |  |  |
| Chromatin Remodeling | Alt | 66 (33.8) | 0.6974 |  | 49 (38.0) | - | 0.7241 |  | 17 (25.8) | - | 0.2209 |
|  | WT | 129 (66.2) |  |  | 80 (62.0) |  |  |  | 49 (74.2) |  |  |
| *ARID2* | Mut | 8 (4.1) | 0.8573 |  | 2 (1.6) | - | 0.6826 |  | 6 (9.1) | - | 0.9838 |
|  | WT | 187 (95.9) |  |  | 127 (98.4) |  |  |  | 60 (90.9) |  |  |
| *KMT2C* | Mut | 8 (4.1) | 0.0517 |  | 2 (1.6) | 12 | 0.0034 |  | 6 (9.1) | - | 0.2487 |
|  | **WT** | **187 (95.9)** |  |  | 127 (98.4) | 80 |  |  | 60 (90.9) |  |  |
| *KMT2D* | Mut | 5 (2.6) | 0.7156 |  | 3 (2.3) | - | 0.4359 |  | 2 (3.0) | - | 0.7163 |
|  | WT | 190 (97.4) |  |  | 126 (97.7) |  |  |  | 64 (97.0) |  |  |
| *EZH2* | Gain | 19 (9.7) | 0.5421 |  | 16 (12.4) | - | 0.7064 |  | 3 (4.5) | - | 0.4695 |
|  | WT | 176 (90.3) |  |  | 113 (87.6) |  |  |  | 63 (95.5) |  |  |
| JAK-STAT | Alt | 53 (27.1) | 0.0836 |  | 42 (32.6) | - | 0.4 |  | 11 (16.7) | 23 | 0.0178 |
|  | WT | 142 (72.8) |  |  | 87 (67.4) |  |  |  | 55 (83.3) | 95 |  |
| *STAT3* | Mut | 11 (6.2) | 0.1674 |  | 12 (9.3) | - | 0.1845 |  | - | - | - |
|  | WT | 184 (93.8) |  |  | 117 (90.7) |  |  |  |  |  |  |
| *STAT3* | Gain | 12 (6.2) | 0.1628 |  | 8 (6.2) | - | 0.9934 |  | 4 (6.1) | 16 | <0.0001 |
|  | WT | 183 (93.8) |  |  | 121 (93.8) |  |  |  | 62 (93.9) | 92.5 |  |
| *JAK1* | Gain | 7 (3.5) | 0.4241 |  | 7 (5.4) | - | 0.4813 |  | - | - | - |
|  | WT | 188 (96.5) |  |  | 122 (94.6) |  |  |  |  |  |  |
| *JAK1* | Alt | 18 (9.2) | 0.5698 |  | 16 (12.4) | - | 0.6671 |  | 2 (3.0) | - | 0.5458 |
|  | WT | 177 (90.8) |  |  | 113 (87.6) |  |  |  | 64 (97.0) |  |  |

Abbreviations: WT, wild-type. Mut, mutation. Alt, alteration. HBV, hepatitis B virus. HCV hepatitis C virus. NBNC, non-B non-C.

**Fig S1 Mutational landscape of HCC patients in the Taiwanese population**

Patients were sorted according to sex and viral infection history. Each column represents one patient. The alteration frequencies of the corresponding genes are shown on the left panel. Genetic alterations were exhibited and ranked according to the occurrence of the pathways. On the right panel, alterations are subcategorized into mutations and copy number variations (CNVs). The significance of the association between genetic variation and sex was evaluated using fisher's exact test or chi-square test, as appropriate. TMB, tumor mutation burden. HBV, hepatitis B virus. HCV hepatitis C virus. NBNC, non-B non-C.
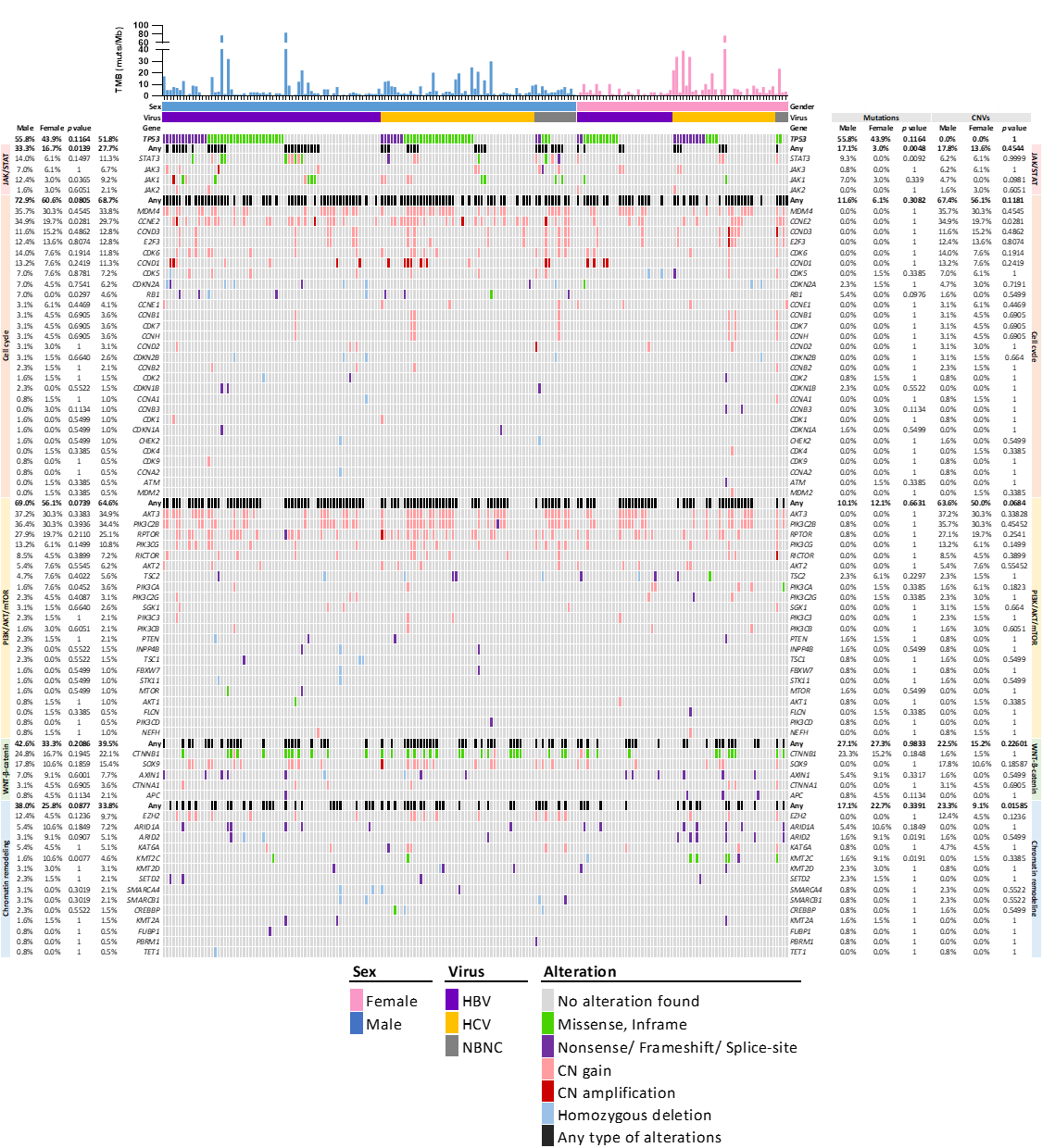


**Fig S2 Tumor mutation burden (TMB) in male and female HCC patients in the Taiwan population, stratified by hepatitis infection history and smoking status**

(A) Comparison of TMB between patients with and without hepatitis B or C infection history. (B) Comparison of TMB between smokers and non-smokers. HBV, hepatitis B virus. HCV hepatitis C virus. NBNC, non-B non-C.


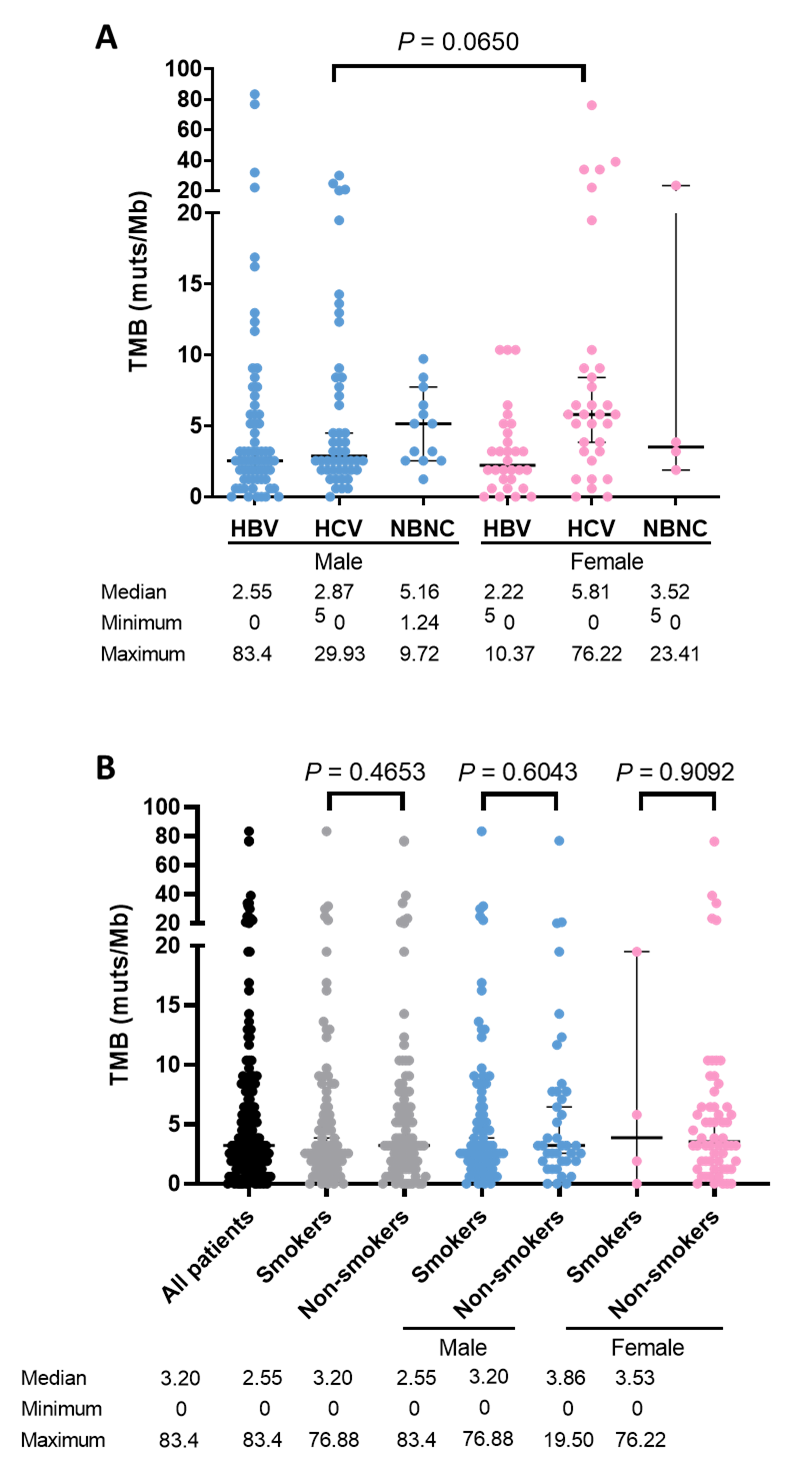


**Fig S3 Tumor mutation burden (TMB) in male and female HCC patients in the TCGA dataset, stratified by hepatitis infection history and smoking status**

(A) Comparison of TMB between patients with and without hepatitis B or C infection history. (B)Comparison of TMB between smokers and non-smokers. HBV, hepatitis B virus. HCV hepatitis C virus. NBNC, non-B non-C.


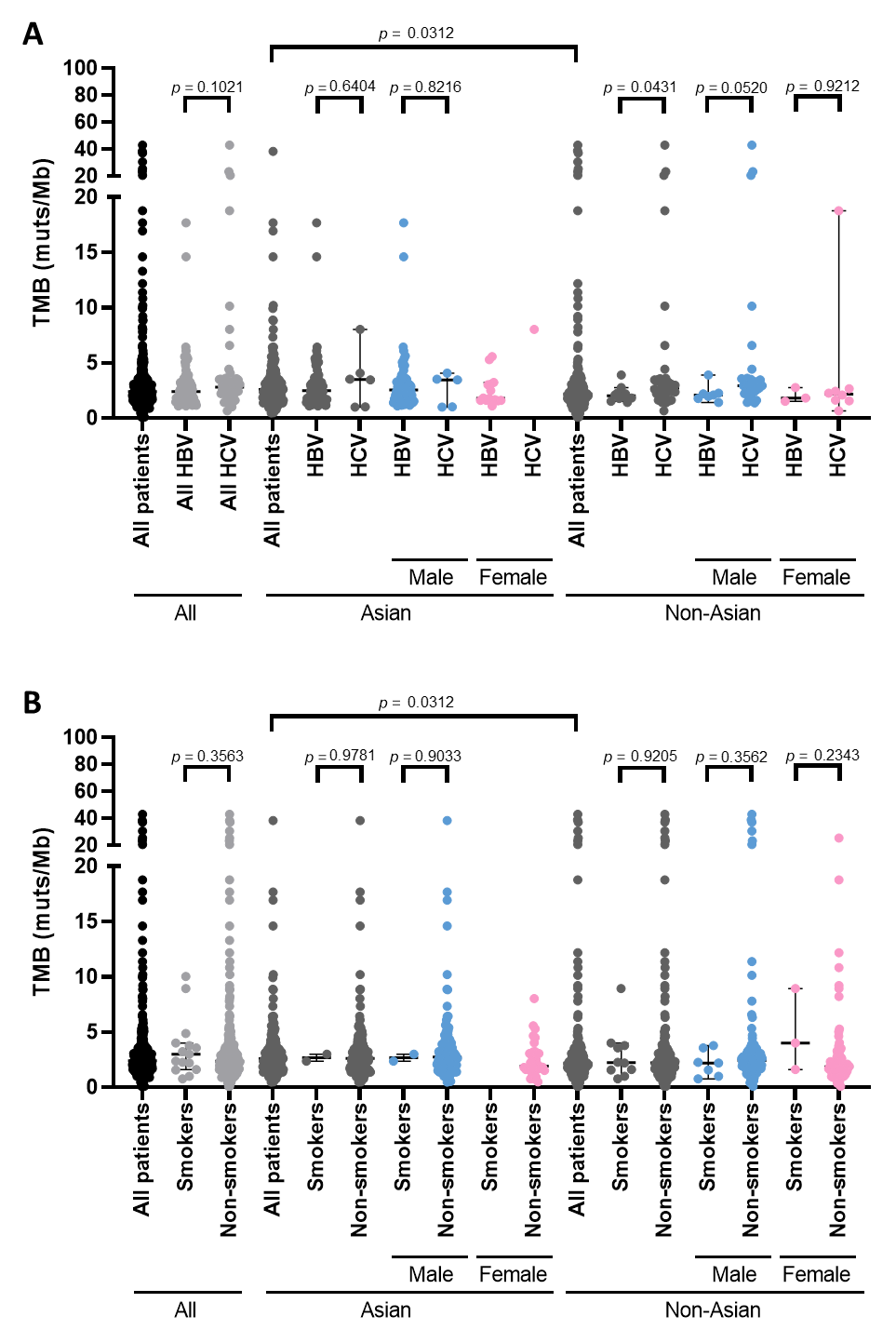


**Fig S4 Distribution of mutational signatures in our studied cohort**

The count represents the number of patients with each mutational signature


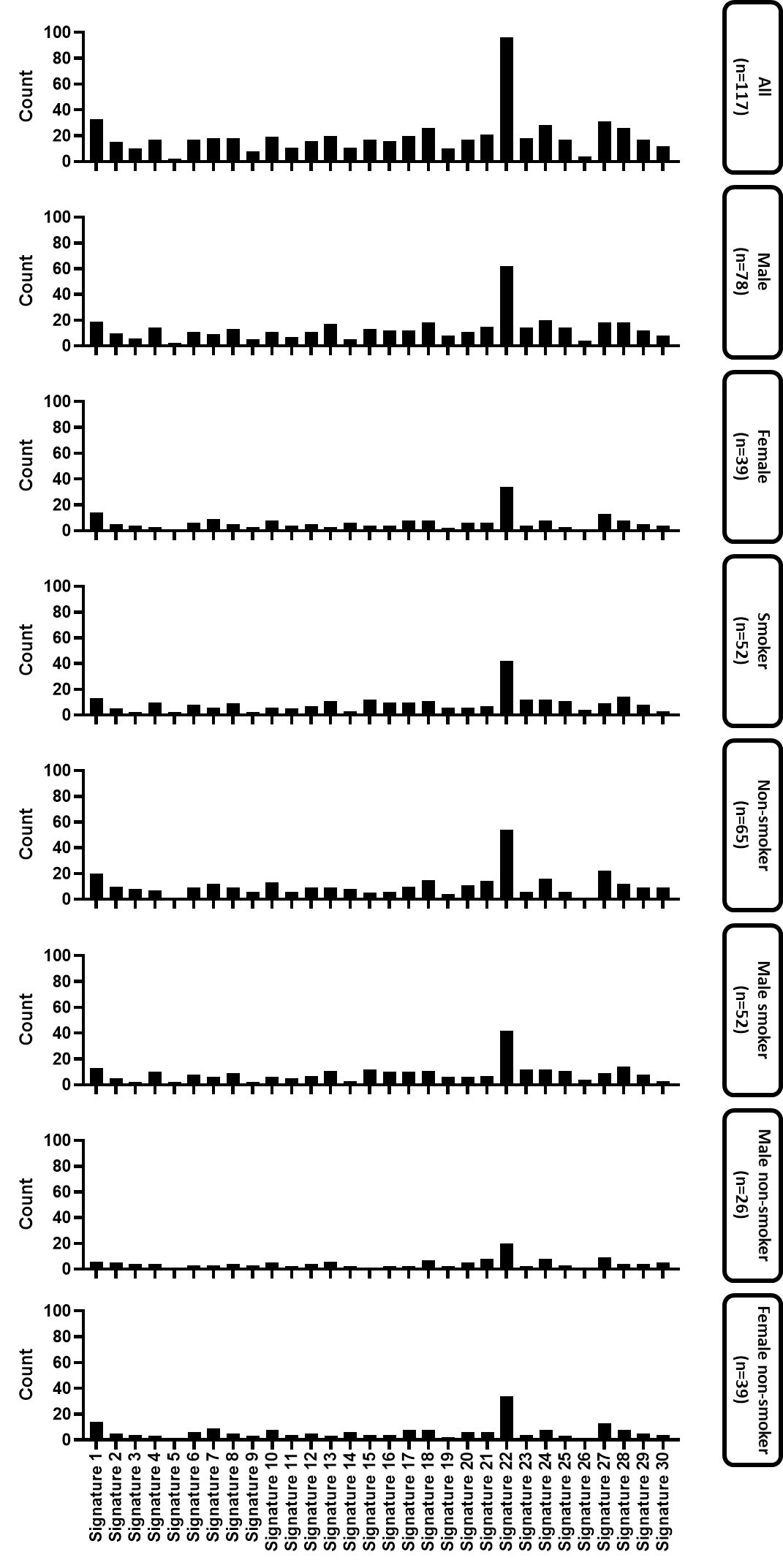


**Fig S5 Lollipop plots illustrate the distribution and mutation profiles of the *JAK1* gene in different cohorts**

The upper image illustrated protein domains and amino acid positions of specific mutations detected in our cohort. The vertical axis showed the frequency of appearance of each mutation. The middle and lower plots illustrated the frequencies of the corresponding mutations in the TCGA Asian and non-Asian subgroups.


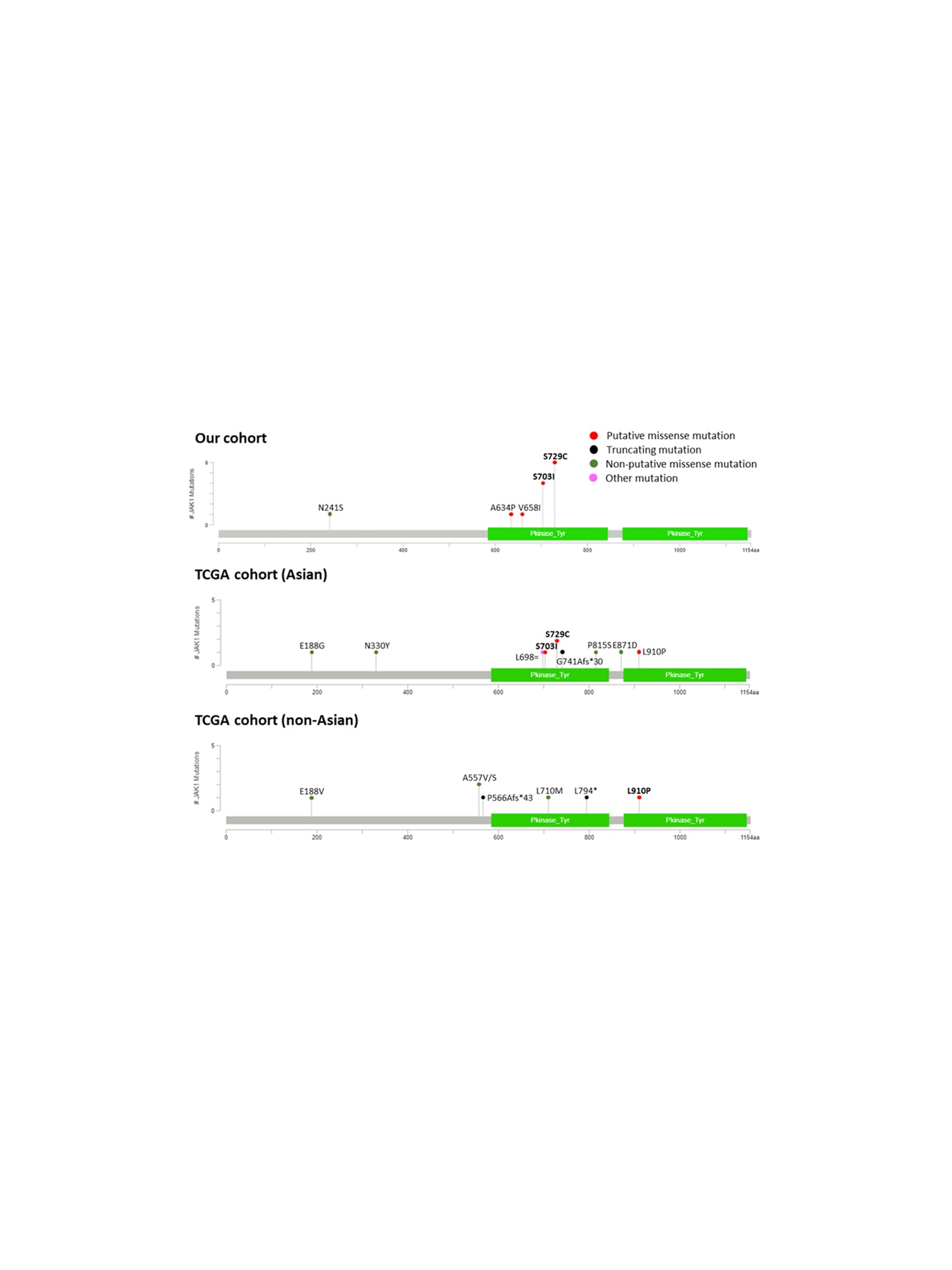


**Fig S6 Lollipop plots illustrate the distribution and mutation profiles of the *STAT3* gene in different cohorts**

This upper image illustrated protein domains and amino acid positions of specific mutations detected in our cohort. The vertical axis showed the frequency of appearance of each mutation. The middle and lower plots illustrated the frequencies of the corresponding mutations in the TCGA Asian and non-Asian subgroups.


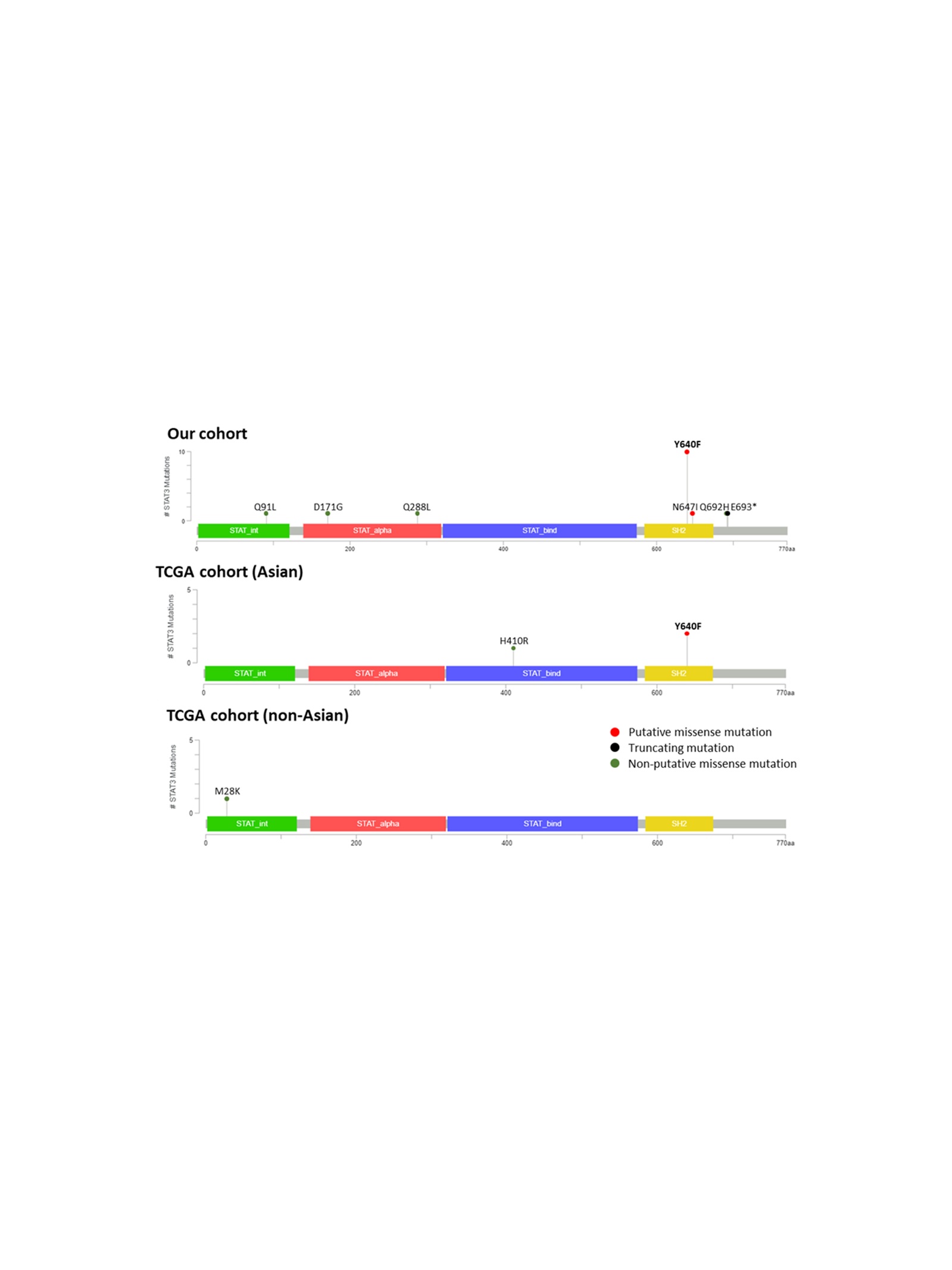


**Figure S7 Kaplan-Meier survival analysis for HCC patients in the Taiwanese cohort**

The overall survival (OS) was assessed for all patients (a, d, and g), male patients (b, e, and h), and female patients (c, f, and i). the analysis is based on the genomic status of the *KMT2C*, *APC*, and *STAT3* genes.


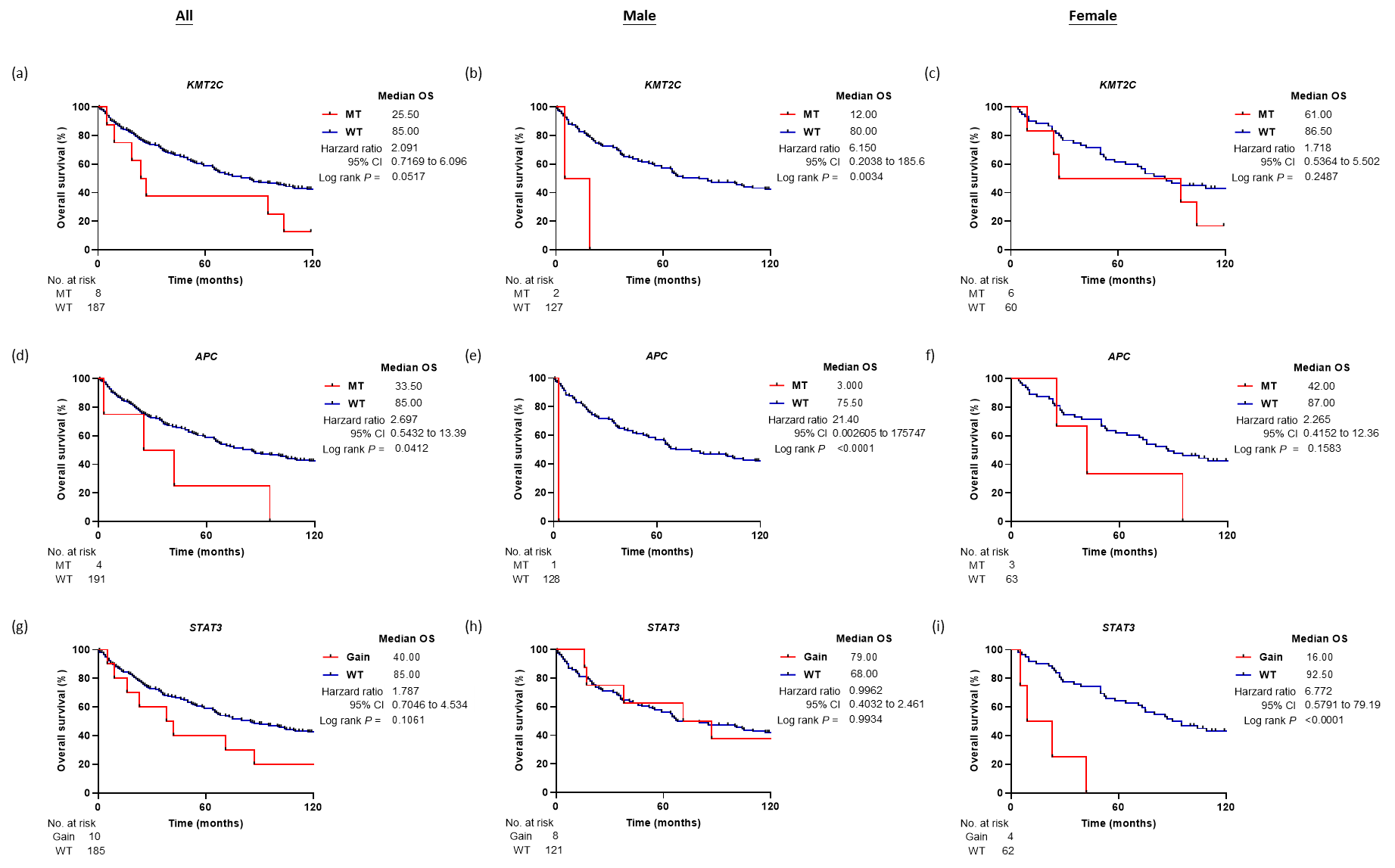


**Figure S8 Kaplan-Meier survival analysis for HCC patients in the TCGA Asian subgroup**

The overall survival (OS) was assessed for all patients (a, d, and g), male patients (b, e, and h), and female patients (c, f, and i). the analysis is based on the genomic status of the *KMT2C*, *APC*, *KMT2C*, and *STAT3* genes.


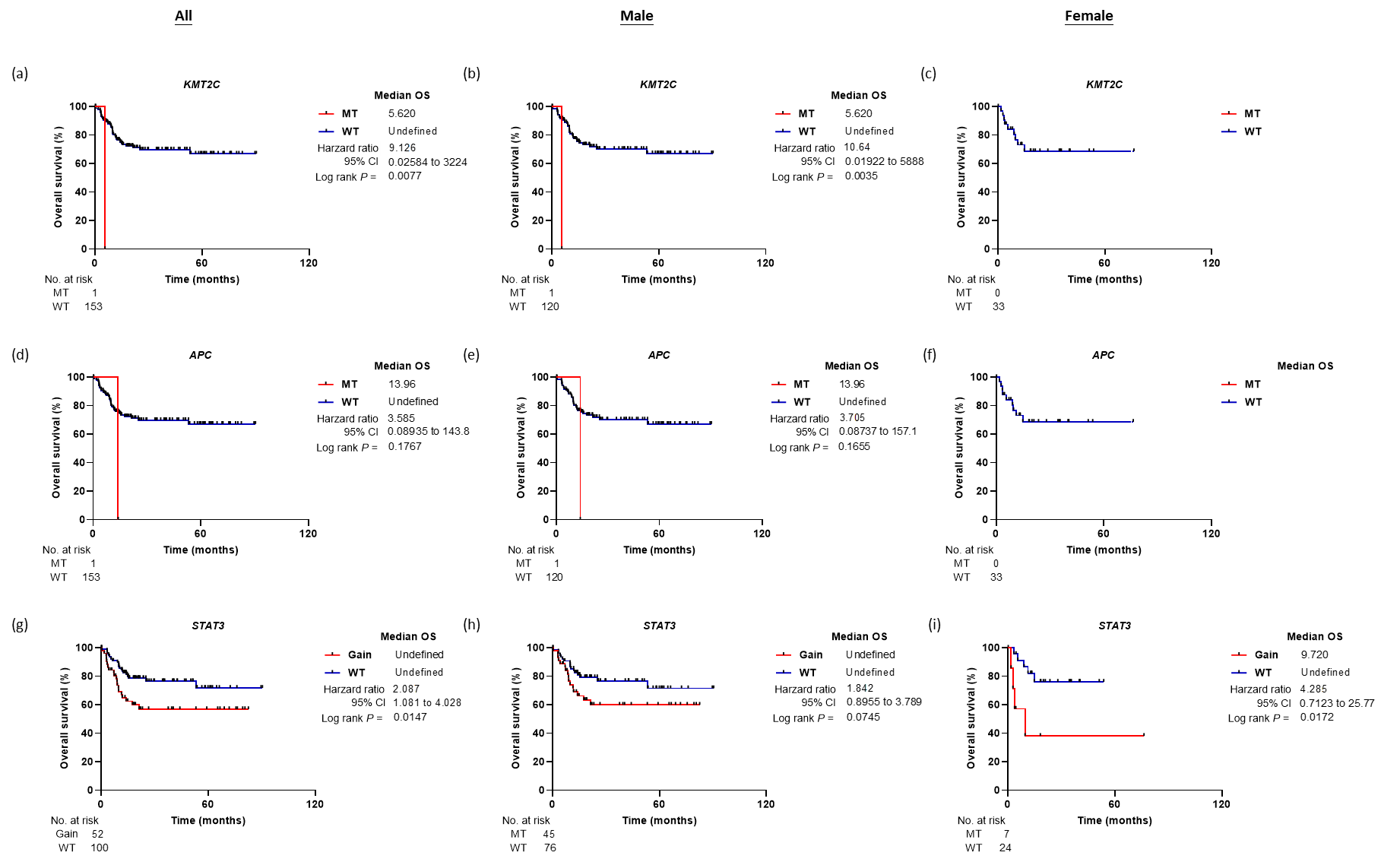

Supplement: Supplementary file 1 — (DOCX 1356 KB) [file 12672_2024_1131_MOESM1_ESM.docx]
